# SMART-BARN: Scalable multimodal arena for real-time tracking behavior of animals in large numbers

**DOI:** 10.1126/sciadv.adf8068

**Published:** 2023-09-01

**Authors:** Máté Nagy, Hemal Naik, Fumihiro Kano, Nora V. Carlson, Jens C. Koblitz, Martin Wikelski, Iain D. Couzin

**Affiliations:** ^1^ Department of Collective Behavior, Max-Planck Institute of Animal Behavior, Konstanz, Germany.; ^2^ Centre for the Advanced Study of Collective Behavior, University of Konstanz, Konstanz, Germany.; ^3^ Department of Biology, University of Konstanz, Konstanz, Germany.; ^4^ MTA-ELTE Lendület Collective Behavior Research Group, Hungarian Academy of Sciences, Budapest, Hungary.; ^5^ MTA-ELTE Statistical and Biological Physics Research Group, Eötvös Loránd Research Network, Budapest, Hungary.; ^6^ Department of Biological Physics, Eötvös Loránd University, Budapest, Hungary.; ^7^ Department of Ecology of Animal Societies, Max-Planck Institute of Animal Behavior, Konstanz, Germany.; ^8^ Department of Zoology, Faculty of Science/Graduate School of Science, Kyoto University, Kyoto, 606-8502, Japan.; ^9^ Department of Migration, Max Planck Institute of Animal Behavior, Radolfzell, Germany.

## Abstract

The SMART-BARN (scalable multimodal arena for real-time tracking behavior of animals in large numbers) achieves fast, robust acquisition of movement, behavior, communication, and interactions of animals in groups, within a large (14.7 meters by 6.6 meters by 3.8 meters), three-dimensional environment using multiple information channels. Behavior is measured from a wide range of taxa (insects, birds, mammals, etc.) and body size (from moths to humans) simultaneously. This system integrates multiple, concurrent measurement techniques including submillimeter precision and high-speed (300 hertz) motion capture, acoustic recording and localization, automated behavioral recognition (computer vision), and remote computer-controlled interactive units (e.g., automated feeders and animal-borne devices). The data streams are available in real time allowing highly controlled and behavior-dependent closed-loop experiments, while producing comprehensive datasets for offline analysis. The diverse capabilities of SMART-BARN are demonstrated through three challenging avian case studies, while highlighting its broad applicability to the fine-scale analysis of collective animal behavior across species.

## Teaser

A multi-sensory tool allows animal collective behavior, interactions, and communication to be studied in large-scale environments.

## INTRODUCTION

The study of animal behavior in naturalistic environments is essential to understand how individuals acquire and process sensory information and use this to make ecologically relevant decisions. The inherent complexity associated with these studies, however, especially over the relevant spatial and temporal scales in which natural behavior is elicited has considerably limited the scope of many behavioral studies. Within the laboratory, behavioral research tends to be conducted in very (often unrealistically) simplified environments that are small relative to that over which many natural behaviors of the species in question are evoked. A further commonly used simplification is to restrict observations made to one, or at most only a few, individuals simultaneously, although many animals, including most model organisms (e.g., mice, rats, zebra finches, and pigeons), exhibit sophisticated social lives.

In recent years, however, there has been a growing realization that these limitations can severely restrict insight into important natural behaviors and that there is an urgent need to develop tools that allow researchers to measure the behavior of multiple animals simultaneously (*1*, *2*). This has both driven and been driven by major technological advances, especially in machine learning (*3*), that allow behavior to be studied in ways not previously feasible. For example, deep learning provides a powerful means to track and identify multiple individuals [e.g., idtracker.ai (*2*) and TRex (*4*)], to estimate time-varying body postures [e.g., DeepLabCut (*5*), DeepPoseKit (*6*), SLEAP (*7*), LMT (*8*), and DANNCE (*9*, *10*)], and via subsequent analysis, quantifying increasingly sophisticated behaviors in a range of ecological contexts (*11*, *12*), including foraging (*13*), prosociality (*14*), aggression and territoriality (*15*, *16*), and reproduction (*17*).

These software solutions also allow the fine-scale kinematics of animals’ motion and/or postures/poses to be related to other streams of data, such as contemporaneous acoustic (*18*, *19*) or neural (*20*) recordings. Appropriate experimental design and methodology is crucial, however, as indoor environments (usually limited in space, volume, and complexity) can be divorced from conditions that give rise to the behaviors themselves and may even cause the animals to exhibit a limited set of behaviors (*21*, *22*). Furthermore, methods typically used to study behavior and perception (e.g., gaze tracking) often require animals to wear comparatively large devices (e.g., helmets equipped with cameras for gaze reconstruction) (*23*). Together, these restrict natural behaviors, for example, by limiting the number and types of interactions that can be exhibited among animals.

Outside the laboratory, technological advancements have also greatly affected the study of behavior (*24*, *25*). Field biologists use ever smaller, and more sophisticated, “biologgers” (devices attached to individuals) for tracking animal movement. The miniaturization, reduction in cost and improved energy efficiency of tags using global positioning system (GPS) and inertial measurement system, allows animal behavior to be studied in natural environments across the globe (*26*, *27*). Analysis of inertial measurements, on board many contemporary biologgers, can also inform scientists about some aspects of behavior, including wingbeats in birds (*28*), the gaits of mammals (*29*), and, in some cases, head posture (*30*, *31*), as well as other context- or species-specific behaviors (*32*). Nonetheless, these data are inherently much less rich than can be obtained in the laboratory environment.

Overall, behavioral studies tend to have been conducted at two extremes of scale; in small, but highly controllable, laboratory environments or in largely uncontrollable, natural environments. Relatively little work, by contrast, has attempted to develop the tools to study behavior at intermediate “mesoscopic” scales [although see (*33*, *34*) for exceptions]. Thus, methodologies that provide a valuable bridge between conventional laboratory and field-based work are generally missing. A short review of recent publications (*8*, *9*, *20*, *22*, *34*–*37*) (Table 1) shows that various groups have proposed experimental setups to study behavior traits of animals in different indoor settings. So far, however, it has been difficult to overcome many limitations such as localization and identification in large areas with many animals, achieving multisensor integration, making precise three-dimensional (3D) measurements, etc., despite the potential for integrating various methodologies to do so.

**Table 1.** Comparison of the capabilities of the SMART-BARN to other studies and systems. Here, we provide a summary of the capabilities of existing setups reported in scientific studies (*8*–*10*, *20*, *22*, *34*–*37*) as compared to the SMART-BARN. A more detailed summary is given in the Supplementary Text. RFID, radio frequency identification; RGB, red-green-blue; RGBD, red-green-blue-depth.

Paper sysyem			Nourizonoz *et al*. (*20*) (Etholoop)	Weissbrod *et al*. (*35*)	Bala *et al* (*36*) (OpenMonkeyStudio	Sarel *et al*. (*34*) Ulanovsky	de Chaumont *et al*. (*8*) (Live Mouse Tracker)	Ballesta *et al*. (*37*)	Dunn *et al*. (*9*) (DANNCE) Direction Aligned Neural Network for Computational Ethology	Anisimov *et al*. (*22*)	SMART-BARN
Sensors (recording + tracking)	Audio		—	—	—	Yes (recording)	—	—	—	Yes (tracking)	**Yes (tracking)**
	Camera	RGB	Yes (behavior)	Yes (tracking)	Yes (markerless tracking)	—	Yes (RGBD-tracking)	Yes (tracking)	Yes (markerless tracking)	—	**Yes (markerless tracking)**
		Infared	Yes (tracking)	—	—	—	—	—	Yes (tracking)	—	**Yes (tracking)**
	RFID		—	Yes (tracking)	—	Yes (tracking)	Yes (identification)	—	—	—	**—**
	IMUM		Yes	—	—	—	—	—	—	Yes	**—**
	Markers/Implants		Yes	Yes	—	Yes	—	Yes	Yes	Yes	**Yes**
	External Sensor Integration		Compatible	—	—	Neural (Recording)	—	—	—	—	**Compatible**
Tracking Features	3D	Position	Yes	—	Yes	—	Yes	Yes	Yes	—	**Yes**
		Posture	—	—	Yes	—	Yes	—	Yes	—	**Yes**
	2D	Position	Yes	Yes	Yes	Yes	—	—	—	—	**Yes**
		Posture	Yes	—	Yes	—	—	—	—	—	**Yes**
	Identity		Yes	Yes	Yes	Yes	Yes	Yes	—	Yes	**Yes**
	Behavior		Yes	Yes	Yes	Yes	Yes	Yes	Yes	Yes	**Yes**
Closed-loop (real time)			Yes	—	—	—	Yes	Yes	—	—	**-**
No. of species reported			2	1	1	1	1	1	4	1	**4**
No. of individuals reported			1 to 3	10	2	2	4	5	1	4	**1 to 20**
Accuracy			<8 mm	80 mm (RFID), 5 mm (RFID + Video)	67 mm (RGB)	90 mm	—	10 mm	6 mm (RGB)	—	<1 mm (mo-cap), 9 mm (markerless)*, 100 mm (acoustic)
Size in meters (length × width × height)			1.7×2×3.1	1.2×1.2×0.8	2.5×2.5×2.8	135×2.3×2.3	0.5×0.5×0.8	1.5×2×2	—	0.6×0.6×0.5	**14.7×6.6×3.8**

*The markerless tracking feature of the SMART-BARN is shown in (*40*).

**Fig. 1.**
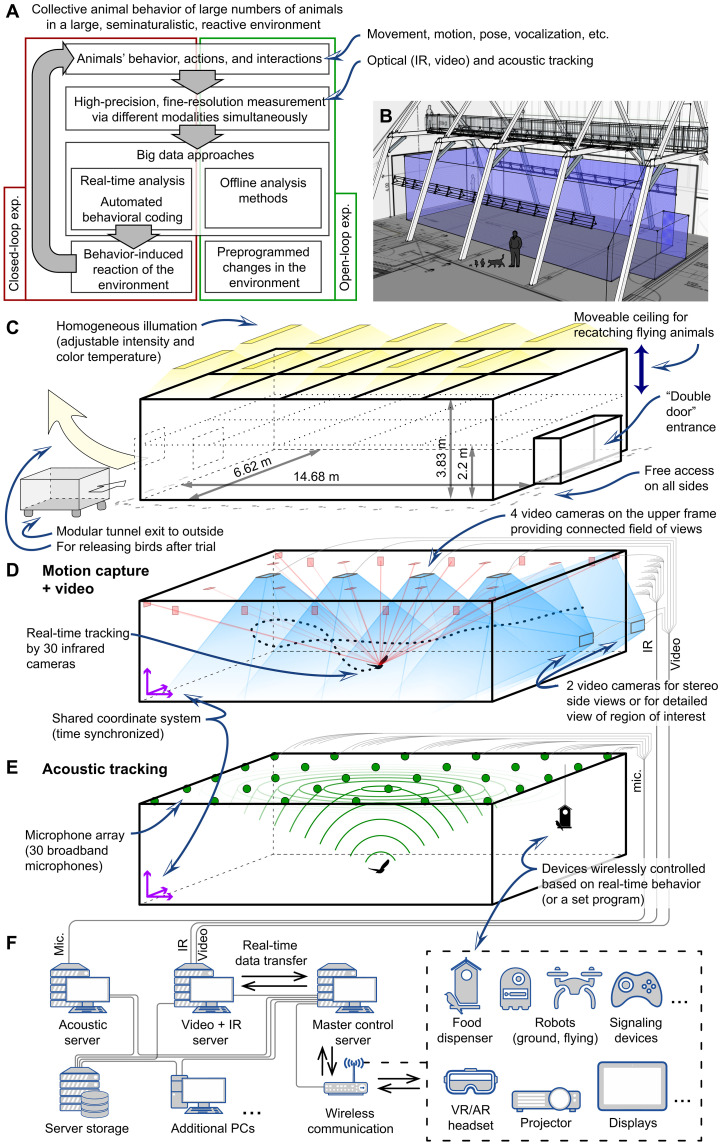
Overview of SMART-BARN and its capabilities. (**A**) Diagram of the main features of the SMART-BARN. (**B**) An illustration showing the experimental area delimited by the net (surfaces in blue) with human and animal silhouettes indicating the scale. (**C** to **E**) Diagram of the tracking volume showing the hardware components and their functionalities in an axonometric view. (C) The nontracking experimental hardware components (lights, frame, net, entryway, and modular tunnel) that create the space where short- or long-term experiments can be conducted on a large variety of species, including domestic, laboratory-bred, or wild-caught animals. (D) Vision-based tracking (visible-light and IR) components. Blue regions illustrate the fields of view of the video cameras. Red rays indicate the IR tracking (mo-cap) provided by triangulation from overlapping views of several cameras for each location in the volume. The mo-cap tracking (dotted curve showing a resulting flight trajectory) could be used for real-time applications (feedback control, virtual/augmented reality, etc.). (E) Acoustic tracking and additional experimental devices. The acoustic tracking uses an array of 30 microphones (locations indicated by green dots) with a frequency range of 100 Hz to 130 kHz. The acoustic data (illustrated as green arcs) can be recorded and localized within the tracking area, as well as used for more traditional acoustic analysis (e.g., spectral analysis). The entire system can accommodate additional real-time wireless–controlled devices, such as robotic units [robots (including flying), food dispensers, etc.] and signaling devices (displays, projections, speakers, haptic-feedback, etc.) that could have preprogrammed behavior or could work reactively on the basis of the behavior of the experimental animals (including group behavior). (**F**) Overview of the connections between the sensors, servers, and wireless-interactive units. VR/AR, virtual reality/augmented reality.

**Fig. 2.**
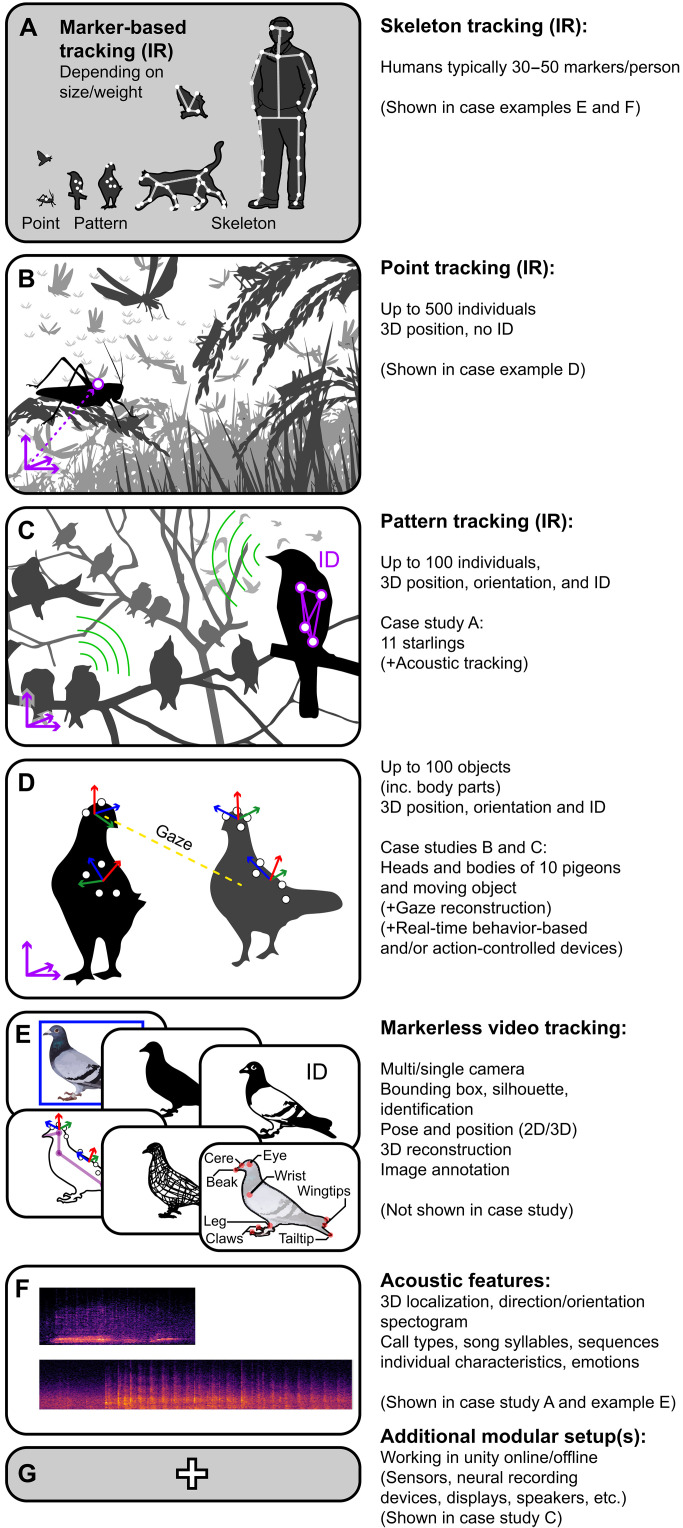
Overview of the modalities that can be captured by the SMART-BARN. (**A**) Marker-based tracking for different animals depending on their size and how much weight they can carry. Markers are depicted as white dots on silhouettes. Connections between markers marking articulated body parts are illustrated with semitransparent white line segments that could produce full skeletons. (**B**) Single marker tracking (marker of 5 mm in diameter and 0.5 g in weight) can be used to get a 3D position for up to 500 small animals (without ID). (**C** and **D**) Marker pattern tracking can be used to obtain the 3D location, orientation, and identification of up to 100 individually recognizable animals or uniquely defined body parts. (C) By using a unique marker pattern on a rigid object (e.g., backpacks used in case studies A and C), individual identities can be maintained and in addition to 3D position, 3D marker orientation can be recorded as well. (D) By attaching multiple unique marker patterns to an individual’s body (e.g., backpack and head markers in case study B), 3D position and orientation of individual body parts can be quantified. Among other possibilities, this allows for the detection and measurement of behavior and perception (e.g., by tracking a pigeon’s head position and orientation, we can reconstruct their gaze). (**E**) Marker-based tracking can be used to produce accurate and (semi)automated annotations for marker-less computer vision by training existing methods and developing new techniques. (**F**) Illustration of spectrographic information that can be obtained from the microphone array. (**G**) Additional modular setups can be included or created by combining multiple parts of the system together.

Our focus here is the development of an integrated system that facilitates the study of animal behavior at these mesoscales (Figs. 1 and 2 and figs. S1 to S5). The requirements are to be broadly applicable to many species, to be modular—allowing it to be deployed in diverse contexts and to allow it to be readily be scaled in size—to provide real-time (or near-real-time) data acquisition to allow closed-loop experimentation (Fig. 1A), and to provide highly accurate 3D positional (Fig. 2, A to E), postural, and acoustic information (Figs. 1, C to F, and 2F) for a large number of animals, simultaneously. We demonstrate the capabilities of this system—SMART-BARN (scalable multimodal arena for real-time tracking behavior of animals in large numbers)—in a large (14.7 m by 6.6 m by 3.8 m) environment (built in a local barn; Fig. 1B) equipped with motion capture system [mo-cap; cameras with sensitivity in the infrared (IR) light spectrum], video cameras (Fig. 1D), acoustic sensors (Fig. 1E), and multiple remote-controlled interactive units (e.g., automated feeders, haptic feedback units, etc.) making it capable of tracking at 300 Hz at submillimeter precision (Fig. 1F). To demonstrate the utility of SMART-BARN, we present three challenging avian case studies in detail and three other example cases briefly, each of which highlights key features that may be desirable for behavioral researchers independent of their specific study species (figs. S6 to S11). The system is used with no substantial change in its parameter settings throughout the different case studies and examples, and, although not a focus of our presented studies, it is therefore straightforward to implement experiments involving multiple species.

1) In case study A, “long-term 3D tracking and bioacoustic analysis of flocking starlings” demonstrates how highly detailed tracking can be achieved for groups of animals over long periods of time (here, for 4 days), allowing for natural, unrestricted interactions between individuals with automated individual identification and localization of their vocalizations.

2) In case study B, “collective attention in pigeon flocks” demonstrates how motion capture and visible-light video systems can be used to obtain fine-scale body movements, including 3D head position and orientation, allowing reconstruction of the visual fields of all individuals in a group, simultaneously, while maintaining their identities.

3) In case study C, “closed-loop experiments with additional sensor integration” demonstrates how the above real-time tracking capabilities can be combined with custom external automated remote-controlled modules for closed-loop experimentation (i.e., an environment that responds to individuals’ behavior) including learning facilitated by remote controlled haptic (vibrating) units on animals and automated food dispensers. Being able to create closed-loop systems can allow a more naturalistic approach to behavioral training, as well as generally providing tools that can greatly increase the scope of behavioral experiments.

4) Case examples D to F show, more briefly, some capabilities of our system not covered in the previous case studies (A to C), such as demonstrating different ecological contexts, modalities, and taxonomic groups. Case example D showcases how tracking can be applied even to very small animals (e.g., insects) that, although often too small for individual identification, still provide highly accurate movement data (e.g., tracking flight behavior or tracking hundreds of individuals in swarms). Case example E showcases the ability to study the biomechanics of behavior, including during interactions between individuals. This is exemplified by tracking the body posture (“skeletons”) of flying bats combined with the contemporaneous analysis of ultrasonic vocalizations. This demonstrates how researchers can address how physical properties of movement are connected to the properties of the sound produced and whether the echolocation signal is deliberately altered, or modified, such as to avoid jamming, when individuals fly together as opposed to alone. Last, by using full skeleton and body tracking of humans, case example F demonstrates the ways in which detailed movement analysis can be combined with other social or experimental conditions to better understand aspects of attention, social interactions, social relationships, and decision-making.

## RESULTS

### Setup

SMART-BARN aims to measure behavior of multiple individuals’ simultaneously in complex, seminaturalistic environments. It is a modular system allowing for flexibility in setting up the environment itself (i.e., the volume of the experimental area) and selecting the instrumental setup (i.e., the tracking/monitoring systems used within; Fig. 1, B to E, and fig. S5).

### Experimental area

The physical environment (tracking area) in which we developed SMART-BARN is an open area surrounded by a frame with fixed length and width (length, 14.68 m; width, 6.62 m; volume, 372 m^3^) but a movable height (2.2 to 3.83 m) to allow for both experimental environment manipulations and to aid in recapturing flying animals (Fig. 1, B to E). This environment is designed to facilitate the study of a wide variety of wild and captive animals (fulfilling animal ethics, laws, and regulation standards, for example, using hormone-free paints, providing durable but collision-safe netting, nonflickering lights, etc.). The “ceiling” of the tracking area is suspended from the ceiling of the structure in which it is housed in and instrumented with both visual and acoustic sensors. The tracking area is surrounded from all sides by a net (10-mm mesh; 0.8-mm strand width; removable) to keep animals within the volume and can incorporate objects (e.g. to create realistic environment or as in one of the example different types of barriers) as it suits the species and/or to allow specific experimental questions to be addressed. The experimental volume supports high-precision light control (including visible, IR, and ultraviolet range) that can be programmed to emit light in specific spectra (e.g., natural light), levels (e.g., dawn), and scheduled times (e.g., following current daylight timing). This allows for comprehensive manipulation of the light environment.

### Sensor arrangement

SMART-BARN includes several subsystems for different modalities of tracking: motion capture (for IR marker–based 3D object tracking), video (for computer vision–based 2D-3D object tracking), and a microphone array with digital acquisition (for 3D acoustic tracking; Fig. 1, D and E). Each subsystem can be used independently that should be required or in concert with the others (i.e., the coordinate systems of all subsystems are temporally synchronized and calibrated to achieve measurements in a single, common coordinate system; see Materials and Methods). The system is also designed to allow the inclusion of additional modules (preprogrammed or interactive units; e.g., audio playback via speakers, compute-controlled feeders and robotic units, projectors or other displays, addition of other lighting sources, etc.) with real-time data transfer between the tracking system and the interactive units (based on automated identification of behavior or interaction).

### Motion capture system (IR)

Optical-IR tracking is widely used for 3D tracking of humans (posture, behavior, activity, etc.) for medical applications, for sports science research, and in the entertainment industry (*38*). We used a commercially available Vicon motion capture system consisting of 30 IR cameras (26 Vero and 4 Vantage; Fig. 1D, table S1, Materials and Methods, and Supplementary Text for more details) which are capable of capturing and providing real-time location data at a high frame rate (up to 300 Hz) using passive (retroreflective) or active markers. For a 6-mm-diameter spherical retroreflective marker (0.5 g), a 3D position with 0.3-mm precision can be obtained (see more details in Materials and Methods).

Single markers allow point tracking (Fig. 2B) by providing a 3D position (for example, the location of one small insect, example D) but each marker is visually indistinguishable from others. Marker positions can be overlaid on video for verification and can also be used for calibration (to provide ground-truth 3D positions, such as to train deep neural networks to achieve subsequent marker-less video-based tracking) and/or for visualization purposes.

Pattern tracking (Fig. 2C) allows individual identification; by creating fixed and unique “patterns” of at least four markers (with each marker at least 2 cm apart in our setup, but this can be reduced, if needed, by adding additional cameras), individualized patterns can be tracked, allowing individual ID and the 3D orientation of the pattern to be determined (case studies A to C). The accuracy is very high allowing multiple patterns to be tracked simultaneously as long as marker patterns are sufficiently distinctive. As above, these data can be spatially and temporally synchronized with video streams.

A further possibility is skeleton tracking that allows 3D tracking of articulated objects when markers are placed on key locations (i.e., joint positions and limbs; Fig. 2, A and D; examples E and F). It is possible to create templates for different species and to customize templates to track multiple individuals simultaneously. Because of the high spatial and temporal accuracy, skeleton tracking proves valuable in the study of biomechanics and behavior.

A considerable advantage of motion capture, be it point tracking, pattern tracking, or skeleton tracking (see also table S2), is that the 3D coordinates can even be estimated for some body parts to which markers are not attached (such as using 3D vectorial information relative to the markers; see gaze measurement in case study B) and these data and/or the locations of the markers are accessible in real time even for very high frame rates and are therefore ideal for closed-loop experiments.

### Video recording and tracking

For visible-light video streams, the SMART-BARN uses six commercially available video cameras (Vicon, Vue, and RGB) that can record at up to 100 Hz and are designed to be synchronized (in both spatial coordinates and time) with the motion capture system. Four of these cameras are mounted on the ceiling to cover the tracking area with a top-down view, and two additional cameras are placed strategically, depending on the experiment being conducted, to provide complimentary views, (e.g., stereoscopic side views or zoomed-in view for regions of interest; Fig. 1D). While not as fast as motion capture, it is still possible to use these video streams for image-based tracking. As noted above, a particular advantage of integrating both motion capture and video is that the former can be used to provide the training data required for the latter allowing researchers to begin with marker-based approaches but to subsequently compliment or even replace them with marker-less tracking (fig. S14) such as computer vision solutions for acquiring avian posture from multiple birds at interactive rates (*39*, *40*) or other deep learning solutions developed by the community (*5*–*7*, *9*).

### Acoustic recording and tracking

For acoustic tracking and recording, the SMART-BARN uses a custom-built system consisting of 30 microphones fixed to the top frame of the netted volume. The microphones have a broadband frequency response ranging from 100 Hz to 130 kHz and are thus able to record both audible and ultrasound sounds (Fig. 1E; case study A and example E). The sample rate can be adjusted to the species of interest (e.g., 100 kHz for birds and 300 kHz for bats). This system allows for long-term continuous recordings allowing standard bioacoustic processing and analysis of all sounds (vocalizations, mechanical sounds, playback of recordings, etc.) and acoustic localization.

Acoustic localization is performed in postprocessing using custom-written Python routines and allows to determine the position, via triangulation, of a sound source in 3D based on the time of arrival differences (TOADs) of a signal at the 30 microphones (see more details in Materials and Methods). The localization accuracy is in the range of decimeters, which typically allows identification of the individual/object that produced the sound (see also fig. S13). Sounds (timing, location, and, if applicable, directionality) can be merged with the data of the motion capture system to have a time-synchronized common global coordinate system.

### Case studies and examples

#### Case study A: Long-term 3D tracking of groups

**Fig. 3.**
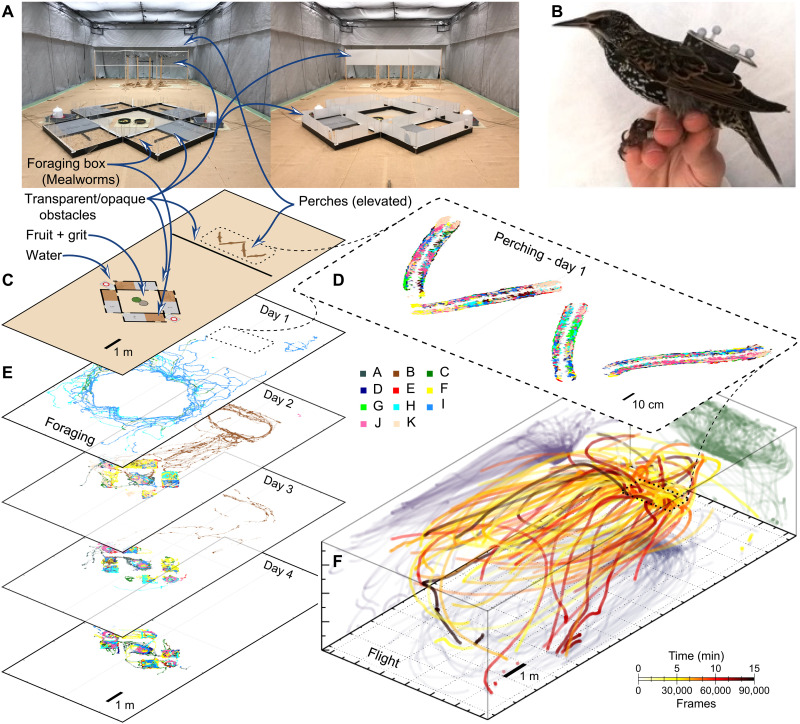
Example for case study A: Long-term (4 days) 3D tracking of a group of 11 starlings—the setup and spatial aspects. (**A**) Two photos of the SMART-BARN showing the additional setup elements for case study A: four perches (at the back), four foraging boxes (filled with mealworms) arranged around additional food bowls (containing fruit and feed), and water on either side. The perches and foraging area were separated by a physical barrier (transparent or opaque). (**B**) Photo of a starling wearing the tracking backpack (with marker pattern). Note that the center of the pattern is further back from the center of the mass of the bird. (**C**) Floor plan of the setup elements to indicate the locations [matching 3D view as (D) to (F)]. (**D**) *X*-*Y* locations of the birds when they were stationary (between heights of 0.5 and 3 m) made from a 2-hour-long recording from the first day (individual identity shown as different colors matching the subsequent panels). The located positions are congruent with the shape of the perching branches, and ID locations are found on both sides of the perch since the location of the marker pattern is off-center. (**E**) Foraging for each bird during the 2-hour recording segment over the 4 days (individuals indicated by colors, *X*-*Y* locations). (**F**) 3D visualization of a 15-min-long segment showing only flight (*v* > 1 m/s) color-coded on the basis of the time (white, yellow, red, and black). 2D orthographic projections are also shown in each plane as single color shadows (green, blue, and purple).

**Fig. 4.**
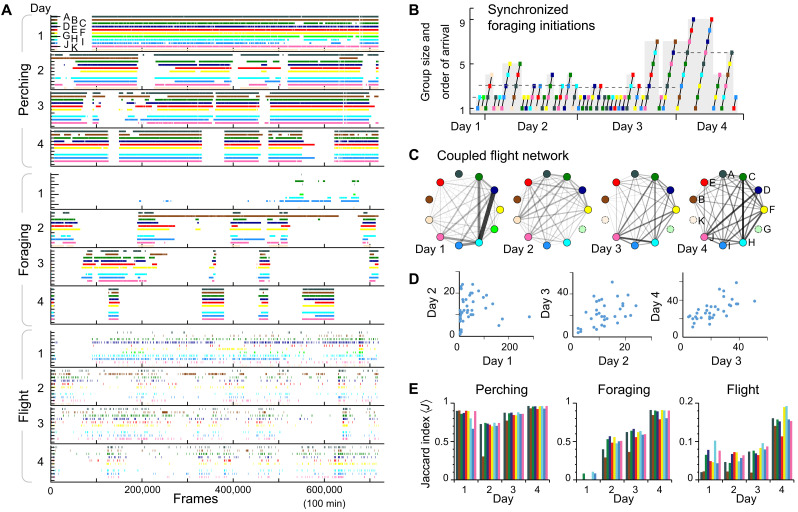
Case study A: Temporal aspects of the behaviors. (**A**) Output of an automated behavioral analysis showing three categories: perching (top), foraging (middle), and flight (bottom). The graph shows the same 2-hour-long recording segment over 4 days for each bird (indicated by colors and separate rows) as Fig. 3. (**B**) The starting times of foraging behavior were analyzed using a Δ*t* = 10-s threshold, and if consecutive birds arrived within 10s, then they were considered as starting their foraging together. These events are shown in the order of the starting time. The local order of individuals changed dynamically, but over time, the individuals became more synchronized and foraging events contained an increasing number of individuals (dashed line shows the daily averages, and gray shading indicates the group size). (**C**) Tracks from flights were analyzed using a directional correlation with delay method (*47*). Networks (constructed separately for each day) illustrate the amount of highly correlated segments (HCSs) obtained using this method (i.e., when two individuals fly together on a very similar 3D path). Flight behavior was found to vary widely, with some birds frequently flying together, while others not. Birds with no connections (shown in dotted circles on days 2 to 4) damaged their marker patterns and so could not be tracked. (**D**) Scatter plot of the HCS [shown in (C)] between consecutive days. (**E**) Behavioral similarity was calculated for each pair, using Jaccard index for each behavioral category [shown in (A)]. Individual scores were calculated averaging over the nine individuals measured throughout all 4 days.

To demonstrate how SMART-BARN can be used to track multiple individuals in a group at very high temporal and spatial resolution, within a large 3D volume, over multiple days, while maintaining individual identity, in conjunction with the microphone array to record and localized sounds produced, we present here sample data from a research project where we tracked a flock of 11 wild caught European starlings, *Sturnus vulgaris*, for 4 days (Figs. 3 and 4 and movie S1). We chose starlings for this experiment as they tolerate laboratory conditions well, are highly social and highly vocal, and spend time perching, flying, and foraging on the ground, thereby making 3D tracking particularly important. Here, when describing the cases studies, we concentrate on the technological and methodological aspects, and we only give a short overview of the scientific goals of the example projects (i.e., we aim to demonstrate how this technology can be used to ask a wide range of scientific questions, depending on researchers’ interests).

Many species live, travel, and make decisions in groups, and to understand how animal groups function, it is necessary to not only understand how individual behavior affects group behavior but also understand how group behavior affects individuals. In addition, for many species, vocalizations are an important part of group movement and decision-making and can shed light on how individuals affect group behavior (*41*). However, to date, because of technological and logistical restrictions (*21*, *22*, *42*), studies aiming to establish how individuals influence one another and make decisions have largely focused either only on movement data often relying on either tracking a limited number of individuals within a group [GPS (*43*)] or recording the use of specific resources by the majority of the group [pit-tags (*44*)]. While there are a few notable alternative solutions [e.g., videos based on tracking of short trajectories of birds in starling murmurations (*45*), 3D tracking in laboratory (*33*, *34*), and the KMP (*46*)], technology that can track multiple individuals, while maintaining individual group members’ identities, over longer duration (in days) in larger areas (in meters) with low localization error (in millimeters) in 3D, and simultaneously capture information about other aspects of social behavior that influences group behavior (e.g., individual vocal behavior and vocal localization) does not exist. This has limited our understanding of how individuals in groups mediate social interactions and decisions based on social, physical, and vocal relationships. The following results are intended to show how synergistic benefits can be obtained by integrating multiple recording modalities.

We used motion capture to track the starlings in 3D as they moved and interacted with one another (Fig. 3, A to F). Each individual was equipped with a small, lightweight (4.5 g) backpack attached via leg loops with a unique marker pattern to maintain individual identity (Fig. 3B). The tracking volume was set up into two zones: the perching zone, with 1.5-m-tall natural branch perches at one end, where the birds spent the majority of their time, and the foraging zone, with food (grit feed and fresh fruit and vegetables), water, and four foraging boxes filled with sawdust and baited with live mealworms to encourage the starlings to engage in natural foraging behaviors and make collective foraging decisions (Fig. 3, A and C). The microphone array (Fig. 1E) recorded all vocalizations during the 4 days and facilitated acoustic localization of calls/sounds after experiments were completed, using custom-written Python routines (code provided with explanation in the Supplementary Materials).

We defined three behavioral categories using 3D trajectories: perching (above 0.5 m and below 3 m, speed of <1 m/s; Fig. 3D), foraging (position below 0.5 m; Fig. 3E), and flying (position above 0.5 m, speed of >1 m/s; Fig. 3F). We tracked 9 of the 11 birds across all 4 days. Two birds damaged their backpacks on days 2 and 3, respectively, so we have incomplete data for those individuals. The precision of the tracking system in action is indicated by bird locations as shown in Fig. 3D, as stationary, off-ground positions are congruent with the shape of the perching branches.

Spatial and temporal patterns of foraging behavior show daily changes in group foraging behavior with birds becoming more directed and synchronized as the days progressed (Figs. 3, D and E, and 4). We defined a “synchronized foraging initiation” as an event when individuals start to forage together [i.e., when difference in time of arrival (Δ*t*) between two consecutive birds was below 10 s]. While only a few individuals made sporadic “foraging trips” in the first days over time, both the timing (Fig. 4A) and the spatial (Fig. 3E) aspects of foraging among birds became increasingly synchronized and thus correlated. Although individuals’ order in arrival to the foraging area varied dynamically, foraging initiations became more frequent and included an increasing number of individuals (i.e., group size increased across days, Pearson’s ρ = 0.390, *N* = 39, *P* = 0.014; Fig. 4B).

We analyzed the coordination of individuals while flying using a directional correlation with delay method (*47*). Using pairwise calculations, we identified highly correlated segments (HCSs; i.e., two individuals flying on similar 3D paths at a similar time) allowing us to construct social networks of flight behavior. Coordination of flight varied widely across days, indicating that some birds flew together often, while others exhibited looser flight associations (Fig. 4C). Those that flew together become more consistent in how much time they flew together over time, as shown by the increasing Pearson correlations of HCSs across days (day 1 versus day 2: ρ_1–2_ = 0.107, *N* = 54, *P* = 0.441; day 2 versus day 3: ρ_2–3_ = 0.454, *N* = 45, *P* = 0.002; day 3 versus day 4: ρ_3–4_ = 0.861, *N* = 34, *P* < 0.0001; Fig. 4D).

To determine the total increase in synchronicity of the flock over time, we calculated Jaccard index using all behavioral categories for each pair, as well as separately for each category. To characterize an individual’s similarity in behavior to the rest of the population, we calculated the mean of the pairwise Jaccard indices between the focal and all other individuals (using only the nine birds that were tracked over the 4 days). The behavioral similarity increased over time (Pearson’s ρ_all_ = 0.478, *P* = 0.003; ρ_perching_ = 0.408, *P* = 0.013; ρ_foraging_ = 0.950, *P* < 0.0001; ρ_flight_ = 0.759, *P* < 0.0001; *N* = 36; Fig. 4E).

**Fig. 5.**
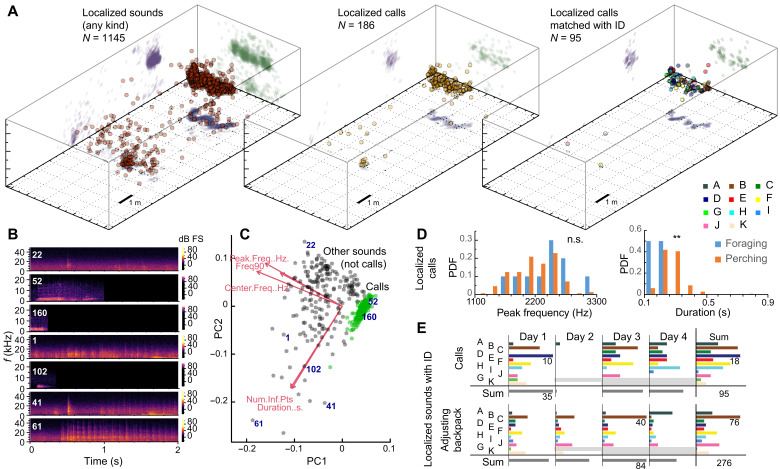
Case study A: Combining motion capture with acoustic analysis and localization. (**A**) 3D visualization of the sound source locations provided by the automated pipeline using a multidimensional optimization. From left to right, all sounds (left), only those that recognized as calls (middle) and those where an individual’s ID could be matched to sound using the timing and location of the sound source and the motion capture data (right; IDs and 2D orthographic projections are shown with colors matching Fig. 3). (**B**) Example spectrograms of recorded sound segments showing the amplitude (brightness of color) of each frequency (in kilohertz) across time (in seconds), allowing for the measurement of the duration and the frequency at which the most power occurs (peak frequency). (**C**) Results of a principal components (PC) analysis for calls (shown with green) and noncall sounds (gray) using spectral properties of the recording; frequency metrics [peak frequency (in hertz), center frequency (in hertz), and 90% bandwidth (in hertz)], temporal metric (duration; in seconds), and one shape measure (number of inflection points). (**D**) Using the location of the sound, we assigned calls to two separate categories: foraging and perching. Shown is the relative occurrence [probability density function, (PDF)] of two spectral properties, peak frequency and duration. n.s., not significant. **P < 0.01. (**E**) Occurrences of localized calls (top) and the sound of manipulating the backpack (bottom) on different days as assigned to individuals. Each part (separated by heavy lines) has a consistent scale, showing the highest values for reference. Gray shading depicts days when individuals damaged their mo-cap pattern (see also fig. S12).

To determine where or which animal produced sounds (calls and noncall sounds), we used acoustic recordings (which had high signal-to-noise ratio of 20.1 ± 4.4 dB, means ± SE). We localized *N* = 1145 sounds (Fig. 5A) using the multidimensional (spatiotemporal) optimization on TOADs (for details, see Materials and Methods). We performed both automated and manual sound analysis on the multichannel recordings (Fig. 5, B and C). Sounds were identified as calls or noncall sounds (including sounds of manipulating the backpack, wing sounds, landing, dropping on the floor, etc.) that were verified by an expert evaluator (N.V.C.).

We performed a principal components analysis on the spectral properties using the loudest channel picked automatically for sound categorization. A total of 16% (186 of 1145) of the sounds were calls, and 90% (1029 of 1137) of all sounds (and 94% of calls; 176 of 186) were produced in the foraging (7%; 13 of 186) and perching (87%; 163 of 186) regions. We recorded few calls during flights, but that is likely due to a relatively low proportion of time spent in flying (Fig. 4A and fig. S12). Calls produced in these two regions had very similar spectral properties, although the call duration was lower in the foraging area [Kolmogorov-Smirnov test, KS = 1.82, *P* = 0.0026; call duration (90%): foraging area, 0.14 ± 0.03 s; perching area, 0.21 ± 0.01 s (means ± SE); Fig. 5D]. Nearly 60% (678 of 1145) of all sounds (and about 50% of calls; 95 of 186) could be assigned to an individual on the basis of the proximity provided by the tracking (fig. S12, A and B). Individuals had large daily variation in the number of calls and backpack-adjusting sounds assigned to them (Fig. 5E), although some individuals exhibited more consistency (e.g., starling B produced the most backpack manipulating sounds 3 of the 4 days).

Tracking multiple individuals and their vocalizations simultaneously within a flock allows not only for an improved understanding of when and with whom individuals choose to coordinate but also for the examination of how multiple modalities contribute to the coordination of individual behaviors within groups. With data that allows for determining not only all individuals’ movements but also their vocal output, it also becomes possible to determine how associations, behavior states, and vocalizations influence individual and group behavior, how individuals use vocalizations and flight initiation to influence the vocal and movement decisions of others, and how other types of technology (e.g., small portable wireless speakers) manipulate the behavior of individuals.

#### Case study B: Collective attention studies in birds using 3D posture tracking

Here, we show how the SMART-BARN’s mo-cap system with the skeleton tracking capability can be used to track fine-scale postural movements of multiple birds in a flock. Specifically, we tracked the positions and orientations of pigeons’ (*Columba livia*) heads and bodies, while simultaneously allowing natural, unrestricted interactions. This allowed us to reconstruct the head-centric views of all pigeons in a flock. We chose pigeons because they are well documented for their visual system (*48*, *49*), neurobiology (*50*), and collective behavior (*51*–*57*).

This case study is part of a larger scientific project, for which we only provide a short overview here. For species that rely on visual perception, including many primate and bird species, the eye is a primary sensory organ from which information is retrieved from the environment. In human and nonhuman primates, the precise measurement of eye movement, namely, eye-tracking, is a frequently used technique to study attention and cognition, such as during perception-action coordination (*58*), social attention (such as gaze following) (*59*, *60*), and anticipation of another’s behavior (*61*, *62*). In birds, although head movement has been frequently used to examine their attentional states particularly in the study of vigilance (*63*), precise recording of eye or head movement has been conducted only recently in the study of natural behaviors, such as navigation (*31*), flocking flights (*64*), and the perception of a male during its courtship display (*23*). However, these technologies still have notable limitations including individuals needing to wear relatively large and/or heavy devices for the species. These limitations often restrict natural behaviors and interactions of individuals and often reduce the number of individuals in a large social group that can be tracked. However, by combining small wearable markers and tracking technology that operates over large areas, thus allowing individuals to interact more naturally, we can remove the majority of these restrictions.

**Fig. 6.**
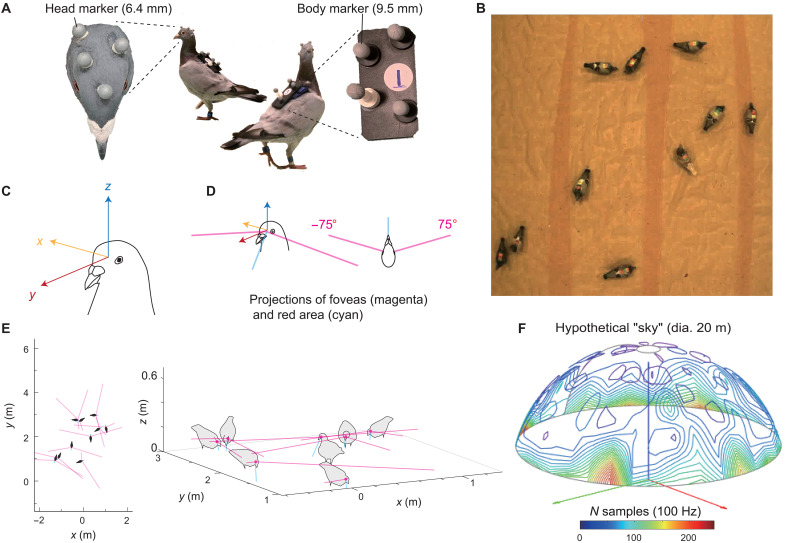
Case study B: Collective gaze tracking in pigeons. (**A**) Pigeons with reflective markers attached to their heads (glued on the feathers) and bodies (glued on a rigid Styrofoam plate attached to a harness). (**B**) View from an over-head video camera filming a flock of pigeons. (**C**) The head-centric coordinate system of a pigeon that is reconstructed from tracking the head. (**D**) Examples of a 3D representation of a head and body with the projections of foveas (in magenta) and red area (in cyan). (**E**) Visualization (still-frame) from the tracking reconstruction of a pigeon flock during free foraging showing two perspectives: as viewed from above (left) and a 3D view (right). Only a part of the volume is shown for better visibility. (**F**) Gaze distribution of a flock during free foraging shown as the sum of all individuals’ gazes projected onto a hypothetical dome. Hotspots (depicted with red colors) indicate locations where a flock as a whole spent more time looking.

We fitted small reflective markers (in unique patterns) to each pigeon’s head and body to record 3D position using the motion-capture system (Fig. 6, A and B). We calibrated the tracking of each pigeon’s head by determining the 3D positions of key morphological points, the eye centers and the beak tip, relative to the centers of the reflective markers using custom head calibration methods and then obtained the head-centric coordinate system for each pigeon with its origin being centered at the midpoint between the two eyes and its *y* axis being parallel to the principal axis of the beak in azimuth and to the horizon in elevation (Fig. 6C). The error of reconstructing the head orientation was estimated as <1° (*65*).

We then constructed the “gaze” of each bird by projecting the bird’s retinal specializations into its head-centric coordinate system: the foveas and red area (Fig. 6D). Pigeon foveas project laterally, at the azimuth of ±75° from the principal axis of the beak and at the elevation of 0° (horizon), and another visually sensitive red area (named on the basis of its appearance in the retina) projects broadly into the lower-frontal visual field (*48*, *49*). Eye movement is relatively restricted in this species, typically within 5° (*66*). A behavioral experiment confirmed that pigeons primarily use the restricted regions around the foveas when viewing attention-getting objects and the frontal visual field when viewing nearby (<50 cm) objects (*67*).

Using this information, we simultaneously reconstructed multiple individuals’ gaze in a flock (Fig. 6, B and E, and movie S2). This setup not only improves previous methods used in avian psychology and sensory ecology but also offers a powerful tool to examine collective attention in groups. To demonstrate this, we visualized how multiple individuals foraging for grains on the ground as a flock distributed their fovea on the hypothetical sky [diameter (dia.) 20 m, the origin being the group centroid, with an azimuth of 0° to 360° and an elevation of 15° to 90°] in a 159-s recording (Fig. 6F). Using this setup and presentation of a model predator, it is possible to examine the fast–time scale dynamics of attention such as during evasion behavior or when exhibiting vigilance. It also becomes possible to ask whether each individual attends to a limited number of conspecifics, how they adjust their behavior and attentional state according to the behaviors and attentional states of neighbors, and how these affect social contagion (*68*), which ultimately shapes collective behavior and attention in groups. Furthermore, combining our gaze-tracking system with the gaze-contingent presentation of a stimulus through real-time stimulus feedback would further improve examination of how information is propagated in a group, while allowing for more fine-scale manipulations of perception.

#### Case study C: Closed-loop experiments with additional sensor integration

SMART-BARN also allows researchers to integrate custom external automated remote-controlled modules that, in combination with the real-time tracking, facilitate closed-loop experimentation in which the environment responds dynamically to the behavior of the animals. Using pigeons as a model system, here, we provide a concrete example of how real-time environmental feedback can be provided to animals depending on the decisions they make. We instrumented birds with remote-controlled haptic (vibrating) devices to provide sensory information as they made movement decisions associated with directional choices toward alternative feeding sites, their reward being dependent on whether they correctly, or incorrectly responded to the haptic feedback (via automated remote-controlled feeders).

**Fig. 7.**
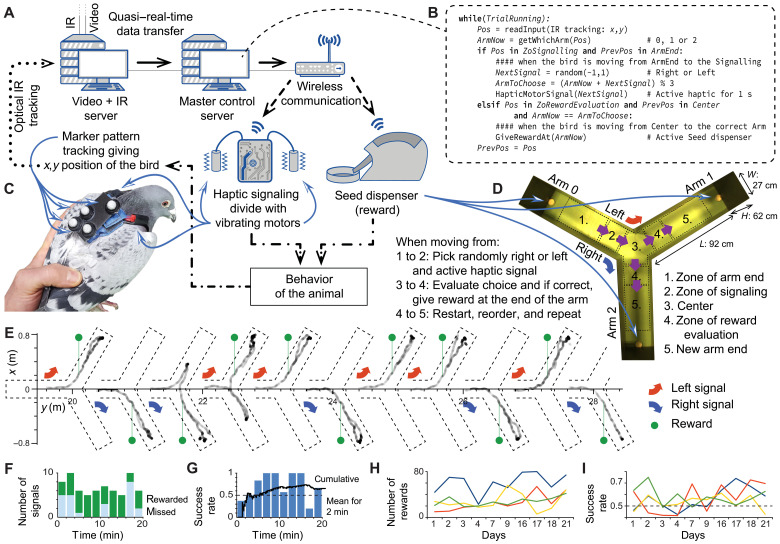
Case study C: Real-time tracking for closed-loop behavioral experiments and operant conditioning. (**A**) Schematic representation of the modules and the flow of information. Pattern tracking gives positional information (top left corner) that is used to automate behavioral identification (follow the arrows clockwise) and behavior-dependent activation of signaling [haptic; more details at (C)] and rewarding devices (three seed dispensers giving seeds if correct behavior was performed). (**B**) Sample of the simplified pseudocode for automated behavioral identification and activation control of the signals and rewards during the Y-maze training. (**C**) Photo of a pigeon wearing a custom fitted backpack with a marker pattern for individual identification and a wirelessly controlled haptic signaling device consisting of vibrating motors are attached to the straps at both shoulders. (**D**) Top down photo of the Y maze with indication of the important zones used for autonomous reward-driven operant conditioning using a haptic signal to indicate which turn should be made to reach the food reward. (**E**) Example trajectory of a bird during a trial shown in a relative coordinate system matching the orientation of the arm in which the signal was provided (each new Y-maze image along the *x* axis shows a new signal provided to the bird). Green pins indicate locations where the turn was evaluated and, if correct, rewards were given. (Note that to avoid rapid multiple signals, if birds turned back in less than 5 s, no new signal was given, and the turn was evaluated neglecting the detour). (**F**) Number of signals (missed and rewarded) pooled for 2-min intervals during a trial. (**G**) Success rate for the same trial indicated by bars and the black curve showing the cumulative ratio. (**H** and **I**) Number of rewards collected (H) and success rate (I) within a trial for four different birds during different days.

**Fig. 8.**
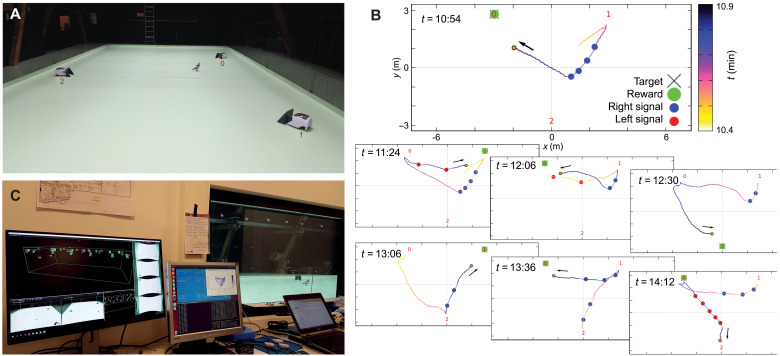
Case study C: Real-time tracking for closed-loop behavioral experiments—showcasing a live experiment. (**A**) Photo taken during the open space experiment showing the pigeon (wearing the backpack) and three wireless seed dispensers. (**B**) Data visualization of the motion of a pigeon, at different time steps, when a feeder was activated (see also movie S3). Circles show the location of the bird when a right (blue) or left (red) turn signal was given (in this trial, controlled by the experimenter). (**C**) Photo taken from the observation room while the system is running live. Through the large window (on the right), the experimenter can observe the experimental arena, while also monitoring each module of the system. All devices can be controlled automatically, as well as manually by the experimenter if needed, via the master control server. Each action is recorded synchronously with the tracking.

Closed-loop systems are an important method not only for training animals (e.g., Skinner boxes) but also to test how changes in the environment, both physical and social, affect individual and group behaviors [e.g. (*69*)]. While many systems currently exist that allow for automated training of individuals during a particular task or in response to a stimulus, they often require animals to engage in behavior outside their natural repertoire and/or context (e.g., pecking a screen) (*70*). By allowing an animal to move freely, while responding in real time to their decision-making, we can study behavior in different scenarios including more naturalistic, and thus ecologically relevant, settings. In case study C (see Figs. 7 and 8), we demonstrate how real-time tracking capabilities can be combined with custom external signals [here, an automated remote-controlled haptic (vibrating) units on animals] to study how a behavior can be facilitated by an induced change in the environment (here, food provided by automated dispensers).

Here, we tested a closed-loop training system in two contexts: a large Y maze (each arm was 92 cm) and in an open environment (i.e., free movement in the full volume of the SMART-BARN). To enable real-time tracking and identification (Fig. 7, A and B), each pigeon wore a backpack outfitted with IR markers in a unique pattern (Fig. 7C). A small radio-controlled haptic device (contained in the backpack; in total of 16 g; Fig. 7C and fig. S15) sent vibration signals to either the left or right side of the pigeon’s body, and a food reward system was implemented in the form of three remote-controlled food dispensers (Fig. 7, B and D). The closed-loop system used the real-time positions obtained from the motion capture pattern tracking to initiate a trial (i.e., when to send a directional cue to the haptic device) and whether to provide a reward to the pigeon (i.e., trigger the automatic feeders), the latter being based on the pigeon’s response to the haptic cue. In this way, the system was able to use the real-time behavioral decisions of the birds to re-enforce specific behavior without manual input.

After a multistep habituation process (see details in Materials and Methods), pigeons gradually learned the task to turn to the correct arm in the Y maze according to the haptic signal, with performance above chance level (cumulative binomial test, of 69 trials, the success rate had *P* ≤ 0.05 for 21 trials, *P* ≤ 0.01 for 12 trials, and *P* ≤ 0.001 for 5 trails; Fig. 7, E to I). For a proof-of-principle trial, we then tested birds in the full volume with three feeders (Fig. 8, A to C). Here, the haptic and reward signal was manually activated by an experimenter via a master control server. An example of a pigeon following the haptic signal to obtain a food reward is shown in Fig. 8B and movie S3.

Being able to create closed-loop experiments, like this, not only allows a more naturalistic approach to behavioral training but also allows for many different types of experimental manipulation as a response to individual behaviors. For example, it is possible to change the visual (e.g., lighting and projections), physical (e.g., access to certain areas and elevation), acoustic (e.g., background noise), and chemical (e.g., release specific scents) environment as individuals interact with it and/or manipulate interactions with conspecifics (e.g., to make an individual move in a particular direction) or in response to heterospecifics (e.g., deploying false predators, etc.). In addition, SMART-BARN could be used to train individuals who can then use that learned behavior when they are in the wild, such as during homing flights in pigeons. For example, individuals could be trained to change heading in response to haptic signals, allowing the investigation of how different individuals in a group influence collective motion (i.e., leader-follower dynamics).

#### Case examples D to F: To demonstrate further possibilities afforded by the SMART-BARN

**Fig. 9.**
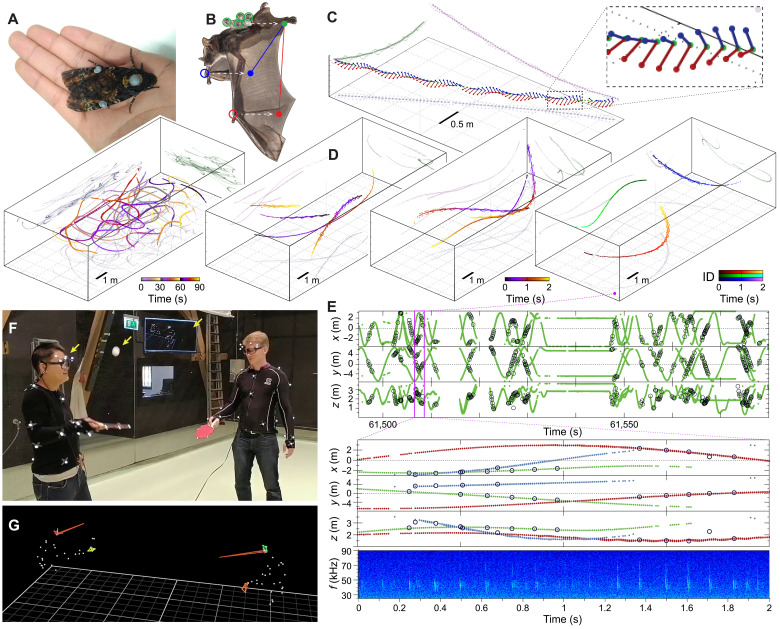
Case examples D to F show additional features of SMART-BARN. (**A**) Photo of a hawkmoth (top; case example D) with markers on the thorax and on the wings. Visualization (bottom) of the tracks of two hawkmoths flying for 90 s (color indicates time). (**B**) Photo of a bat (case example E) wearing a marker pattern attached to its back (highlighted by green circles). During the recording the wrist joints (locations indicated by red and blue circles for left and right, respectively) were marked with flat sticker markers (not shown in the photo). Markers allowed reconstructing the main skeleton, shown with red and blue dots and line segments (shifted to the right for better visibility). (**C**) After postprocessing, the flight mechanics can be reconstructed when the wing markers are tracked successfully. (**D**) Two-second-long examples of four bats. Some bats lost their wrist markers during the trials. (**E**) *x*, *y*, and *z* coordinates shown in time for acoustic localization (black circles) of echolocation clicks and mo-cap tracking (green dots) for longer segment (on top) and a 2-s-long time window (in the middle, color showing ID) that matches the rightmost plot of (D). Example spectrogram (shown at the bottom) from a single microphone channel illustrating the amplitude (hue of color) of each frequency (in kilohertz) in the ultrasonic range across time for the same time window (see also fig. S13). (**F**) Photographic illustration of detailed tracking of humans (case example F) in our setup that can also be used for similar-sized vertebrates. Specialized eye-tracking glasses allowed reconstruction of subject’s gaze in real time as indicated by the screen at the back (see also movie S4). Yellow arrows highlight the location of the wearable eye trackers, the ball, and the screen. (**G**) Visualization of the tracking data. Paddles and glasses are highlighted, and red lines depict the gaze.

In case example D, “moth tracking” shows how the system can track flying insects that can carry a restricted number of markers per individual. This setup uses a point tracking feature that does not allow for individual identification, but tracking of a large numbers of insects is possible with only a single marker on the body of each individual. In addition, we also attached markers to the wings, so wing movements of an insect can be partially tracked, allowing for the collection of data beyond a single position in space for an individual (Fig. 9A and movie S4).

In case example E, “collective movement and coordination of echolocation in bats” demonstrates how the system can be used for mammal species. Skeleton tracking allows us to capture the detail of the wing movement, the most important aspect of the flight. Echolocation calls can be captured by the microphone array the sensitivity of which extends into the ultrasonic range, allowing for future experiments examining aspects of movement and other types of behavior such as how bats avoid signal jamming when flying close to each other (Fig. 9, B to E, figs. S4 and S5, and movie S4).

In case example F, “detailed body movement and gaze tracking for humans” shows the state of the art of skeleton and gaze tracking of humans and thereby demonstrates how our system can be potentially applied to the fine-scale tracking of other nonhuman large vertebrates (Fig. 9, F and G, and movie S4). Moreover, if we combine this human tracking with the other examples and case studies, then our system could readily be used to study animal-human interactions.

## DISCUSSION

Our case studies and examples demonstrate the diverse utility of SMART-BARN, a modular and scalable integrated system for the study of individual and collective behavior. It allows experimenters to investigate a wide range of questions on leadership, vigilance, cooperation, coordination, visual-information transfer, and the role of vocal communication in a group behavior without the typical limitations found in laboratory approaches. For example, it can allow the behavior of a single individual, or of individuals in groups, to be studied in seminatural and, via closed-loop feedback and remote sensors, responsive (interactive) environments. The modularity of the system allows for the integration of new data streams, such as neural recordings and virtual/augmented reality, providing considerable future potential.

Our current setup has several limitations, although we anticipate that these will be resolved by further customization and future technological advances. Critically, the motion-capture system requires animals to be equipped with multiple small reflective markers (to track 3D orientations and individual identities), which is not yet suitable for some small-bodied species (such as *Paridae* species). The motion capture system also has some positional dependency in its performance, and we get the reported tracking accuracy up until around 3.5 m in height (if animals are marked on their back), above which their tracking is lost (because of the geometry of our imaging volume). Moreover, especially when one needs to track the smallest possible patterns over hours, the tracking accuracy of the motion-capture system may deteriorate because of minor changes in environmental temperature and position of motion-capture cameras and damages/detachments of reflective markers. Last, the costs to build the entire system are not small in terms of time and budget, although we expect that they will be reduced in the future, especially with marker-less tracking solutions.

There are a variety of experiments that one could readily perform in the SMART-BARN by a mix-and-match approach from the case studies and examples, in addition to further customization. Specifically, we tracked up to 80 reflective markers in case study B, and with that one could potentially track 80 terrestrial, or flying, small animals with point tracking (e.g., moths; example D). Although, here, we did not test the upper limit of markers that can be tracked simultaneously, we note that it is likely to be far greater than the maximum of 80 presented here (closer to several hundreds based on tests performed by the technical team at Vicon). Thus, researchers who require the study of larger groups may find such a system—or our system, as we invite collaboration—appropriate; although we advise testing with real-world conditions to ascertain the upper limit for the given species and marker combination if individual identification is required. Furthermore, the interactions of multiple animals (including humans) can be investigated in a relatively large volume, for example, in foraging/vigilant pigeons (using skeleton tracking; see case study B), swarming insects (using point tracking), and prey-pursuing predators (using the combination of these). In addition, the real-time tracking ability of the system is capable of controlling drones interacting with one another (or with animals) based on bioinspired behavior principles. As evidenced by our case studies, we anticipate that the SMART-BARN and an equivalent system will facilitate research on a wide range of animal behavior questions at mesoscopic scales.

## MATERIALS AND METHODS

### Setup

In this section, we will provide details regarding the setup of SMART-BARN. The details will mostly include specifications of some of the technologies and a broad overview of the available features.

### Motion capture system: 3D tracking with IR cameras

We deployed commercially available Vicon motion capture system consists of 30 IR cameras: 26 Vero v2.2, (2048-pixel by 1088-pixel resolution) and 4 Vantage V5 (2432-pixel by 2048-pixel resolution), providing data at high frame rate (between 50 and 300 Hz; see table S1) and in real time [typically, the latency is less than 10 ms when using through networking, but Vicon Tracker can process and provide data as low as 1.5 ms (*71*)].

### Motion capture

The system operates with two types of markers: (i) passive markers and (ii) active markers. In most case studies and examples shown here, we used passive markers, but the system was calibrated using a calibration “wand” that has active markers. This allows the cameras to be recalibrated even if animals wearing the passive markers are inside the arena.

The measurements of the mo-cap system can be accessed in two ways: (i) online (real time) via streaming and (ii) offline via storage files. The online modes are used during closed-loop experiments, where tracking results are processed via customized scripts (written in Python; available at https://zenodo.org/record/7890292) and used in the experiment to trigger devices or provide stimuli based on movement of the animal (see case study C). Users can also transmit the data to other wireless devices such as a small sensor, robot, or computing using (arduino unit).

### Video recording (RGB)

3D marker tracking results are visualized in video images through proprietary software (Vicon Nexus) provided by the manufacturer. We have developed a customized software to replicate the process of mapping 3D data over video footage (available at https://zenodo.org/record/7890292). This allows us to create datasets for computer vision applications (*39*, *40*) (see also fig. S14).

### Microphone array

For acoustic recording, the SMART-BARN uses a custom-built system consisting of 30 microphones (Knowles FG-23329). Vocalizations and nonvocalization sounds are amplified using custom-made amplifiers (produced by the Workshop at the University of Konstanz from commercially available parts) and analog-digital converted (ADC) using four National Instruments (NI) ADC cards (NI USB-6356). The four ADC cards were synchronized with each other using a synchronization pulse produced by a master ADC card, and the three remaining cards follow this as slaves. The sample rate can be adjusted to the species of interest, for example, for birds, a sample rate of 100 kHz was chosen, and for ultrasonic calibration, recordings of bats 300 kHz are used. Long-term continuous recordings achieved by storing all audio data (MALTA-Software by “CAE Software and Systems”). Acoustic localization is performed in postprocessing using custom-written Python routines (codes available for future users open source at https://zenodo.org/record/7890239) and allows the determination of the position of a sound source in 3D based on the TOADs of a signal at the 30 microphones.

### Acoustic tracking

Acoustic tracking in our setup works by triangulating a sound source. The position of a sound source (both animals and sound-emitting devices) is computed by measuring the TOADs between the microphones. After band pass–filtering the recordings, an automated detector running on one channel detects acoustic events (or an expert can identify events manually), and the corresponding recordings on the other 29 channels are selected accordingly as detailed later. Because of the strong signal deterioration over distance, especially of high frequencies, usually a subset of channels is automatically chosen for localization. The TOADs of the signal between those channels is measured by cross-correlation. Given the large number of receivers, resulting in an overdetermined array, a relative localization error can be computed by iteratively removing receivers from the analysis. The localization accuracy is in the range of tens of centimeters (for both high and low frequencies), which typically allows identification of the individual/object that produced the sound. Sounds (timing, location, and, if applicable, directionality) can be merged with the data of the motion capture system to have a time-synchronized common global coordinate system.

We assigned calls to regions simply determining where, in space, the calls were (i.e., perching zone, foraging zone, and flying zone; Fig. 3, C to F), and we assigned calls to individual birds using two criteria: (i) The closest individual was within 30 cm, and (ii) the next closest individual was a minimum of 50% farther from the sound than the closest individual.

### Sensor fusion

Our setup consists of three different tracking modalities: IR-based motion capture (mo-cap) for marker-based tracking, RGB video cameras for markerless tracking, and microphone array for acoustic tracking. Data fusion is necessary to combine results from different modalities and make most of the system. This is done with temporal synchronization (data capture rates) and spatial registration.

#### Temporal synchronization

Temporal synchronization is needed to synchronize data collection rates for different modalities in the SMART-BARN. In the existing system, the mo-cap system is already synchronized with video cameras. In addition, mo-cap system supports both hardware and software triggers for adding extra modalities or sensors to the existing setup. These modalities may be used for additional measurements, e.g., inertial measurement units (IMUs) or biologgers or devices that may be used for delivering stimuli for experiment, e.g., screen or projectors. Hardware triggers usually send start-stop signals to other devices with master-slave configuration. It defines when to start taking measurements in sync with the mo-cap system. Software triggers are useful for activating or deactivating units in conjunction with the tracking results of the mo-cap system using LAN (local area network) or wireless-LAN communication protocol. These units may be preprogrammed to deliver stimuli via speakers, projectors or other displays, addition of other lighting sources, etc., or interactive units such as microcontrollers, controlled feeders, or robotic units. Time delays for hardware triggers are minimal and, hence, preferred for setting up measurement devices. In the existing system, temporal sync with acoustic recording is achieved in postprocessing by matching time stamps.

#### Spatial registration

All 3D measurements are registered in the same coordinate system for fusing the data collected from different tracking modalities. Mo-cap manufacturer already offers a calibration protocol for both IR cameras and RGB video cameras (explained below). The calibration protocol is performed to determine positions of the cameras and define a global coordinate system. We chose a center point on the ground plane of the SMART-BARN as a global coordinate system with *x* axis along the long edge, *y* axis along the short edge of the barn, and *z*-axis points upward. The camera positions are used to measure 3D position of objects using computer vision methods (object detection, stereo triangulation, etc.). A custom-defined protocol is used to register the microphone array with the global coordinate system defined by the mo-cap system. The calibration process is based on the same principles for both acoustic and optical systems. For clarity, in the following, we write details of the calibration of the modules separately. First, we explain the calibration protocol of the mo-cap system and then calibration of the acoustic system with the mo-cap system.

### Calibration for mo-cap and video cameras

In our setup, we calibrate the video camera and IR cameras simultaneously. We use the protocol defined by the manufacturer (i.e. Vicon) but the general concept of multicamera calibration is well established in computer vision literature (*72*). An object with known geometry is used as a calibration object, and a sequence of images is captured by positioning the calibration object in different positions and orientations in the target volume. We used Vicon active wand for calibration. It is ideal that multiple (not all) cameras with overlapping fields of view capture the same position and orientation of the calibration object. Once the image sequence is captured, the geometric information of the calibration object is used to compute intrinsic (lens distortion) and extrinsic parameters (position and orientation) for each camera. Extrinsic parameters are essential for triangulating 2D coordinates obtained from multiple images. The extrinsic calibration changes when the position or orientation of cameras is altered; therefore, to achieve this high-resolution tracking as reported in this paper, it is important to recalibrate the system once cameras are moved or bumped by animals. Calibration may also change because of vibrations or changes in temperature. Intrinsic calibration is useful to compute distortion parameters of the lens and to compute a matrix that allows projecting 3D metric information to 2D pixel information using projective geometry. Intrinsic parameters for cameras remain stable and do not change unless lens position, focal length, or aperture properties are changed. Intrinsic parameters do not need to be computed when camera positions are changed.

The typical time required to calibrate the complete system is 20 min including data capture and processing. The calibration time can be substantially reduced when intrinsic parameters are not recomputed. The existing system is designed to provide submillimeter accuracy (*71*). Change in calibration parameters usually results in loss of accuracy over time and may interfere with pattern tracking. Multiple calibrations could be used for postprocessing.

### Calibration of mo-cap with acoustic sensors

We have defined a customized calibration protocol to register the acoustic tracking system with the motion capture system (available at https://zenodo.org/record/7890292). The first step is to calibrate the motion capture system such that it can produce results in a 3D coordinate system. Similarly, the acoustic system is setup in such a way that sound signals can be processed and localized in the 3D coordinate system. A minimum of four measurements of the same object in both the coordinate systems are required to register both the systems. We define a calibration object (a directional speaker) and attach markers to it. We place the calibration object in different positions in the SMART-BARN and turn on the speakers to record short sequences with the mo-cap and acoustic system. The location of the calibration object is static within one sequence, and, thus, for each position, we obtain the 3D position of the calibration object with both mo-cap and acoustic system. Both the systems have very different accuracy, and, therefore, we take more than four positions to get balanced results. The outcome of the registration process is a 6-DOF (degrees of freedom) pose that allows transporting results of the acoustic localization to the global coordinate system defined by the mo-cap system.

### Statistical analysis

Here, we showed complex features of the system through case studies. However, to counterbalance for the complexity in the features, we presented the results based on basic statistical methods, such as Pearson correlation tests, cumulative binomial tests, and two-sample Kolmogorov-Smirnov tests when comparing two distributions. The values for *N*, *P*, and the specific statistical test performed for each experiment are included in the appropriate figure legend or main text.

### Methods: Case study A

#### Subjects

We used 12 wild-caught starlings (*S. vulgaris*) obtained from the Baden-Wurttemberg region. We also provide sample data for a flock of 20 starlings. Birds were kept in aviaries on-site and moved from their home aviaries to the SMART-BARN the evening before the experiment started. They were weighed and had backpacks attached via leg loops before being released into the tracking arena. During evening hours, they had access to night roosts, and, during the day, they only had access to perches in the tracking area. They had ad libitum access to food and water in the SMART-BARN.

#### Ethics

Experiments were approved by the Regierungspräsidium Freiburg under permit numbers Az. 35-0185.81/G-18/91 on starlings. The catching of wild starlings was approved by Amt für Baurecht und Umwelt Untere Naturschutzbehörde under permit number M1900420.

#### Backpacks

We made sure that the tracking backpacks weighed less than 5% of a starling’s average body weight (4.5 g). To keep the backpacks light, we made the bottom, front, and sides out of cellular rubber sheets (LUX Ersatzbelag für Zellgummiglätter Classic 28 cm by 14 cm by 8 mm, LUX) that were glued (Pattex Kraftkleber Classic universeller Kleber; Pattex, Germany) to a 0.2-mm balsa wood top. We created 12 unique ID patterns (see the “Case study A: Long-term 3D tracking of groups” section for specifics) that were printed as 3-mm holes on a replica of the balsa wood top via a 3D printer [PLA (polylactic acid) plastic, 0.35 mm]. To create a 3D pattern on each backpack, we super glued (UHU Sekunden Kleber Blitzschnell Supergel, UHU GmbH & Co. KG, Germany) an M3 plastic screw (1 mm by 8 mm, 3 mm by 5 mm) into each hole. We screwed a 6.4-mm IR marker from OptiTrack (NaturalPoint Inc., DBA OptiTrack, Corvallis, USA) to each screw. Backpacks were attached to the starlings using leg loops (see the “Case study A: Long-term 3D tracking of groups” section for specifics).

#### Tracking arena setup

The tracking arena was enclosed by the PP net (see Supplementary Text) to keep the birds inside and a series of Tyvek panels to keep the birds from seeing beyond the net. Full spectrum lights were synchronized to the current daylight cycle; they changed the light spectrum during dawn and dusk hours to mimic natural light (warmer colors) and kept full spectrum natural light during full daylight hours.

The tracking arena had two zones: a perching zone where birds had access to 1.5-m-tall perches made of natural sticks and a foraging zone with bird feed (Fettfutter extra, Blattner Heimtierfutter, Germany), fresh fruit and vegetables, and water ad libitum, as well as four foraging boxes (93 cm by 165 cm by 11 cm; width by length by height) containing live mealworms (Palmowski and Fauna Tropics, Germany) arranged around the bird feed and fresh fruit and vegetables (Fig. 3C). Access to new mealworms happened twice a day, once in the morning when the foraging boxes were bated and half was covered, and once in the afternoon when the covers were moved to the other half of the foraging box remotely. Husbandry and raising/lowering night perches followed the same schedule across days. Night perches were lowered, paper flooring was replaced, food and water were replaced in the morning before 8:15, new mealworm areas were exposed at 11:15, and night perches were raised at 17:00 daily. There were also these plastic barriers (LEE Filters Worldwide, U.K. transparent clear color filter, no. 130, and opaque white technical filter, no. 220) around the edge of each foraging box (22 cm high) and between the perching zone and the foraging zone (75 cm high starting at 1.2 m), and these were changed out during morning husbandry. Two loudspeakers [Mackie (USA) Thump 51A; frequency range, 32 Hz to 23 kHz; max sound pressure level (SPL), 127 dB; 686 mm by 442 mm by 356 mm] were placed facing each other near the perching zone and the foraging zone out of sight of the birds.

#### Procedure

Before any birds were introduced to the SMART-BARN, we used the Vicon Nexus system to calibrate the cameras. Birds were caught in the aviaries using hand nets and placed in birdbags before being transported from their aviary to the SMART-BARN. Once inside the SMART-BARN, all birds were processed (ID, weight, and assigned backpack) before a backpack was attached and tested for fit. Starlings were then released into the tracking area. Starlings were released in the SMART-BARN the evening before recordings were taken to ensure that they had time to acclimate to the backpacks and their new surroundings before recording commenced. Starlings then lived in the SMART-BARN for 4 days, and, during this time, recordings were taken throughout the day. During the four experimental days, IR and audio recordings were taken continuously from 8:15 to 16:15, and each morning during husbandry (7:30 to 8:00), the cellophane barriers were changed (either dummy change or changed to the opposite type) so the birds experienced both filter types two times in 4 days (transparent: Lee Color Filter 130 Clear, and opaque: Lee Technical Filter 220 White Frost). In addition, during experimental days, birds were exposed to either road noise (two of the four experimental days) at an average SPL of 75 dB or silence (two of the four experimental days) played for 3 hours in the morning (starting at 8:15) and 3 hours in the afternoon (starting at 13:15). Data presented in this paper was only taken from the time the playback was paused mid-day (11:15 to 13:15). After the four experimental days were up, starlings were caught the morning following the final recording day shortly after sunrise using a mist net (we lowered the ceiling so the birds could not escape over the mist net). They were measured again [ID, weight, and condition; no bird lost more than 8 g of their body weight, well within the normal fluctuations during winter—especially as birds were caught in the morning before feeding (*73*, *74*)] before their backpack was removed and they were placed in a bird bag for transport. They were then transported back to their aviary and released.

#### Data analysis

Peak frequency (in hertz) is the frequency at which the most power occurs, center frequency (in hertz) is the frequency at which half of the power in the call falls below, and 90% bandwidth (in hertz) is the difference between the frequency below which 95% of all power occurs and the frequency below which 5% of all power occurs. Inflection points measure the number of inflection points in the contour of the peak frequency measured across the entire sound. We found that calls were relatively similar between all individuals [means ± SD; call duration (90%), 0.18 ± 0.04 s; call bandwidth (90%), 1.71 ± 0.21 kHz; aggregate entropy, 5.36 ± 0.21 bits; peak frequency, 2.12 ± 0.44 kHz; Fig. 5D].

### Methods: Case study B

#### Subjects

Twenty homing pigeons (*C. livia*) bred at the Max Planck Institute of Animal Behavior at Möggingen, Radolfzell (Germany) participated in this study. Birds roost and breed in a loft (five compartments with a size of 2 m by 2 m by 2 m each). Birds had ad lib access to water and were fed grains once a day. They wore custom-made backpacks (made of fabrics and elastic bands, 3 g; Fig. 6). On the day of experiments, they were brought from the loft in a carrier box (60 cm by 35 cm by 23 cm) to the SMART-BARN, and after the experiments (it took typically 1 to 2 hours), we brought them back to the loft and fed them additional grains on the same day.

#### Ethics

The study protocol was approved by the governmental office in Germany (no. 35-9185.81/G-19/107).

#### Apparatus

Before a daily experiment, we attached four reflective markers (dia. 6.4 mm, together <1 g; OptiTrack) on the feathers of a pigeon’s head with double-sided tape and glue (those markers were 1.5 to 2 cm apart from one another). In addition, we attached a Styrofoam plate (3.5 cm by 7 cm by 0.3 cm, width by length by thickness) with four markers (dia. 9.5 mm, Vicon, together <1.5 g) to the backpack with velcro (together <5 g). Those four markers were screwed on marker bases (5 or 10 mm in height), which were glued on the Styrofoam plate (those markers were apart from one another at a minimum of 2 cm; Fig. 6). We used custom-made software (MATLAB) to determine the 3D arrangements of the markers on the Styrofoam plate so that the pattern of arrangement on each individual became as unique as possible (to identify each individual reliably in the motion-tracking system). We removed the head markers and the Styrofoam plates from the pigeons after the daily experiment.

#### Calibration

We reconstructed the head-centric coordinate system based on 3D coordinates of head markers and morphological key points (beak tip and eye centers). We then aligned the coordinates of head markers and morphological key points using either of the two calibration methods that we developed over the course of the study; both calibration methods were comparable in their accuracy, but we critically improved the efficiency of procedures in the second calibration method to reduce the time required for handling pigeons during the head calibration process. In the first calibration method, an experimenter lightly held the head of each pigeon and manually aligned the custom calibration grid with four 9.5-mm markers to the pigeon’s beak tip and eye centers. In the second calibration method, an experimenter lightly held the pigeon’s head in a calibration grid equipped with four standard webcams (C270, Logitech, Lausanne, Switzerland), which were synchronized in a software (MultiCam Capture, Corel, Ottawa, Canada) to capture the pigeon’s head from multiple angles. To give information about an absolute scale (length) to the system, a triangular scale (2 cm on each side) was attached temporarily to the root of the pigeon’s beak during the calibration. We used “Camera Calibrator” in MATLAB (MathWorks, Natick, USA) to correct distortions of captured images. We then manually identified the coordinates of markers and key morphological points in captured images and then reconstructed the 3D coordinates using the structure-from-motion functions in MATLAB (available at https://zenodo.org/record/7890386).

We used the obtained 3D coordinates to construct the head-centric coordinate system with its origin located at the midpoint of two eyes, its *y* axis parallel to the horizon and pointing to the beak tip, its *x* axis parallel to the horizon and pointing to the right eye, and *z* axis orthogonal to *x* and *y* axes. We defined the horizon as the transverse plane of a pigeon’s head in each recording; thus, for each recording, we calculated the single median value of roll angles (around 0°) and that of pitch angles (around −30°) from all frames and then rotated each bird’s head orientations by those median values respectively for roll and pitch in all frames.

#### Data analysis

In a motion-capture software (Nexus 2, Vicon), we defined the bird’s skeleton, which consisted of head and backpack rigid bodies connected with a free joint and then automatically labeled all markers in all frames. Although the head markers were often mislabeled by the software within the head’s rigid body, we corrected this error in our custom MATLAB codes (available at https://tinyurl.com/yj5hfhrt) by first identifying the time frames in which the distance between the head markers abruptly changed, larger than 5 mm in adjacent frames, and then permuting the marker labels in each section until these abrupt changes were eliminated in the entire recording. Last, we converted the global coordinates of visual targets into the local coordinates in the bird’s head-centric coordinate system.

### Methods: Case study C

#### Subjects

Twenty-eight homing pigeons participated in this study, and the details are identical as described in the “Methods: Case study B” section.

#### Ethics

The study protocol was approved by the governmental office in Germany (no. 35-9185.81/G-16/151).

#### Computer-controlled food dispensers

We used three computer-controlled wireless seed dispenser (Treat&Train Remote Reward Dog Trainer, PetSafe) with two modifications: a funnel and a tube guided seeds (safflower seeds selected as their most preferred seed) to a cup; and the hand-held remote controller was connected to a custom-designed electronic board to be able to receive control signals through the parallel port.

#### Haptic feedback and vibration signaling device

We constructed the haptic feedback devices from commercial available radio-controlled modeling components. It contained separate units connected with thin wires (fig. S15): the main board (2.8 cm by 2.8 cm by 0.4 cm, 8 g in weight), two vibration motors (0.7 cm in diameter, 1.5 cm in length, and 1 g in weight), and a battery (E-flite, 3.7 V, 150 mA·hour, 12C LiPo; 4.1 cm by 1.1 cm by 0.6 cm, 2 g in weight). The board and the battery were held in a cloth backpack, and the vibration motors were attached to the straps at the shoulders of the pigeons. In total, the device including the harness weighed 16 g, which is around 3% body mass of a 500-g bird.

#### Computer control for the wireless devices

Three food dispensers and the two vibrating motors of the haptic feedback device were separately controlled through an eight-channel analog radio controller (Robbe-Futaba FC-15) that was connected to the PC’s parallel port using custom-written scripts in Python. The system was modified from a previous study (*75*), available to use.

#### The Y maze

We used an open-top Y maze made out of opaque polyvinyl chloride boards (gray; 5 mm in width), with three identical arms joint at 120°. Each arm was 92 cm by 27 cm by 62 cm (length by width by height).

#### Training protocol

The training protocol was as follows. First, we placed pigeons in the Y maze where seeds were presented on the floor (pretraining 1). Then, we placed pigeons (13 individuals) in the Y maze without seeds presented on the floor (pretraining 2); one arm of the maze was blocked, and no haptic stimuli were given. The pretraining was conducted for 30 min to habituate the birds to the setup, and when birds approached a new arm (as shown by the real-time tracking), the automated feeder provided seeds to motivate them to move between the two arms. Those birds (four individuals) that passed the criteria to receive at least 20 portions of seeds within 10 min in at least three consecutive experimental days were then placed in the Y maze (all three arms open) equipped with the haptic device that provided stimulation, and only received a food reward if they turn in the correct arm signaled by the haptic signal either in their left of right shoulder. Then, the two best performing individuals were used in an open-field test, and one of them followed the signal in this new context.

### Methods: Case examples

#### Example D

We trialed the tracking system using three adult African death’s head hawkmoths (*Acherontia atropos*), reared in a laboratory setting (*76*). We marked the individuals with three markers (one 6-mm spherical marker on the thorax and two 3-mm semispherical markers at the midpoint of the edge of the wings). We recorded tracks at 200 Hz.

#### Example E

We marked four bats (pale spear-nosed bat, *Phyllostomus discolor*) with marker pattern (four pieces of 6-mm spherical) on the back and on their wrist joints (1 flat “sticker” marker each). We recorded tracks at 100 Hz. The study protocol was approved by the governmental office in Germany (no. 35-9185.81/G-21-042).

#### Example F

We present an example of two humans (M.N. and F.K.) wearing a motion capture suit (37 markers, including six markers the glasses) and wearable eye tracking glasses (Tobii Pro Glasses 3, Tobii AB) while playing with a trackable ping pong set (paddles and ball). The study protocol was approved by the University of Konstanz Institutional Review Board, 39/2022.

#### Custom software development

We have developed multiple scripts (specific computer code) to process the data provided by various systems used in the SMART-BARN. All of the implementations are in open git repositories, and all have sufficient documentation.

#### Example application: Automated dataset for machine learning

The scripts we developed to visualize data on video images are useful for more than just visualization. We provide one example of how to use the 3D tracking data from the experiments to get a bounding box (see fig. S14) around the animals. The setup can generate automatically annotated datasets with thousands of images in a reliable and robust manner with high accuracy and minimal manual effort. These datasets are essential for development of markerless motion capture methods for animals. In future, markerless tracking may replace the marker-based tracking or enhance the performance in cases where tracking is lost because of occlusion of markers.

## References

[1] A. Pérez-Escudero, J. Vicente-Page, R. C. Hinz, S. Arganda, G. G. de Polavieja, idTracker: Tracking individuals in a group by automatic identification of unmarked animals. Nat. Methods 11, 743–748 (2014). 24880877 10.1038/nmeth.2994

[2] F. Romero-Ferrero, M. G. Bergomi, R. C. Hinz, F. J. H. Heras, G. G. de Polavieja, Idtracker. ai: Tracking all individuals in small or large collectives of unmarked animals. Nat. Methods 16, 179–182 (2019). 30643215 10.1038/s41592-018-0295-5

[3] N. Vogt, Automated behavioral analysis. Nat. Methods 18, 29 (2021). 10.1038/s41592-020-01030-1 33408390

[4] T. Walter, I. D. Couzin, TRex, a fast multi-animal tracking system with markerless identification, and 2D estimation of posture and visual fields. eLife 10, e64000 (2021). 33634789 10.7554/eLife.64000 PMC8096434

[5] A. Mathis, P. Mamidanna, K. M. Cury, T. Abe, V. N. Murthy, M. W. Mathis, M. Bethge, DeepLabCut: Markerless pose estimation of user-defined body parts with deep learning. Nat. Neurosci. 21, 1281–1289 (2018). 30127430 10.1038/s41593-018-0209-y

[6] J. M. Graving, D. Chae, H. Naik, L. Li, B. Koger, B. R. Costelloe, I. D. Couzin, DeepPoseKit, a software toolkit for fast and robust animal pose estimation using deep learning. eLife 8, e47994 (2019). 31570119 10.7554/eLife.47994 PMC6897514

[7] T. D. Pereira, N. Tabris, A. Matsliah, D. M. Turner, J. Li, S. Ravindranath, E. S. Papadoyannis, E. Normand, D. S. Deutsch, Z. Y. Wang, G. C. McKenzie-Smith, C. C. Mitelut, M. D. Castro, J. D’Uva, M. Kislin, D. H. Sanes, S. D. Kocher, S. S.-H. Wang, A. L. Falkner, J. W. Shaevitz, M. Murthy, SLEAP: A deep learning system for multi-animal pose tracking. Nat. Methods 19, 628 (2022). 10.1038/s41592-022-01426-1 PMC9007740 35379947

[8] F. de Chaumont, E. Ey, N. Torquet, T. Lagache, S. Dallongeville, A. Imbert, T. Legou, A.-M. Le Sourd, P. Faure, T. Bourgeron, J.-C. Olivo-Marin, Real-time analysis of the behaviour of groups of mice via a depth-sensing camera and machine learning. Nat. Biomed. Eng. 3, 930–942 (2019). 31110290 10.1038/s41551-019-0396-1

[9] T. W. Dunn, J. D. Marshall, K. S. Severson, D. E. Aldarondo, D. G. C. Hildebrand, S. N. Chettih, W. L. Wang, A. J. Gellis, D. E. Carlson, D. Aronov, W. A. Freiwald, F. Wang, B. P. Ölveczky, Geometric deep learning enables 3D kinematic profiling across species and environments. Nat. Methods 18, 564–573 (2021). 33875887 10.1038/s41592-021-01106-6 PMC8530226

[10] J. D. Marshall, U. Klibaite, A. Gellis, D. E. Aldarondo, B. P. Ölveczky, T. W. Dunn, The pair-r24m dataset for multi-animal 3D pose estimation. bioRxiv 10.1101/2021.11.23.469743 [Preprint] (2021). 10.1101/2021.11.23.469743.

[11] D. Tuia, B. Kellenberger, S. Beery, B. R. Costelloe, S. Zuffi, B. Risse, A. Mathis, M. W. Mathis, F. van Langevelde, T. Burghardt, R. Kays, H. Klinck, M. Wikelski, I. D. Couzin, G. van Horn, M. C. Crofoot, C. V. Stewart, T. Berger-Wolf, Perspectives in machine learning for wildlife conservation. Nat. Commun. 13, 792 (2022). 35140206 10.1038/s41467-022-27980-y PMC8828720

[12] S. Christin, É. Hervet, N. Lecomte, Applications for deep learning in ecology. Methods Ecol. Evol. 10, 1632–1644 (2019).

[13] R. E. Johnson, S. Linderman, T. Panier, C. L. Wee, E. Song, K. J. Herrera, A. Miller, F. Engert, Probabilistic models of larval zebrafish behavior reveal structure on many scales. Curr. Biol. 30, 70–82.e4 (2020). 31866367 10.1016/j.cub.2019.11.026 PMC6958995

[14] M. J. M. Gachomba, J. Esteve-Agraz, K. Caref, A. S. Maroto, M. H. Bortolozzo-Gleich, D. A. Laplagne, C. Márquez, Multimodal cues displayed by submissive rats promote prosocial choices by dominants. Curr. Biol. 32, 3288–3301.e8 (2022). 35803272 10.1016/j.cub.2022.06.026

[15] A. P. H. Bose, P. Nührenberg, A. Jordan, Female–female conflict is higher during periods of parental care in a group-living cichlid fish. Anim. Behav. 182, 91–105 (2021).

[16] A. Haluts, S. F. G. Reyes, D. Gorbonos, R. I. Etheredge, A. Jordan, N. S. Gov, Spatiotemporal dynamics of animal contests arise from effective forces between contestants. Proc. Natl. Acad. Sci. U.S.A. 118, e2106269118 (2021). 34857634 10.1073/pnas.2106269118 PMC8670459

[17] L. Long, Z. V. Johnson, J. Li, T. J. Lancaster, V. Aljapur, J. T. Streelman, P. T. McGrath, Automatic classification of cichlid behaviors using 3D convolutional residual networks. iScience 23, 101591 (2020). 33083750 10.1016/j.isci.2020.101591 PMC7553349

[18] V. Demartsev, A. S. Gersick, F. H. Jensen, M. Thomas, M. A. Roch, M. B. Manser, A. Strandburg-Peshkin, Signalling in groups: New tools for the integration of animal communication and collective movement. Methods Ecol. Evol. 14, 1852–1863 (2023).

[19] B. J. Hightower, P. W. A. Wijnings, R. Scholte, R. Ingersoll, D. D. Chin, J. Nguyen, D. Shorr, D. Lentink, How oscillating aerodynamic forces explain the timbre of the hummingbird’s hum and other animals in flapping flight. eLife 10, e63107 (2021). 33724182 10.7554/eLife.63107 PMC8055270

[20] A. Nourizonoz, R. Zimmermann, C. L. A. Ho, S. Pellat, Y. Ormen, C. Prévost-Solié, G. Reymond, F. Pifferi, F. Aujard, A. Herrel, D. Huber, EthoLoop: Automated closed-loop neuroethology in naturalistic environments. Nat. Methods 17, 1052–1059 (2020). 32994566 10.1038/s41592-020-0961-2

[21] D. Stowell, L. Gill, D. Clayton, Detailed temporal structure of communication networks in groups of songbirds. J. R. Soc. Interface 13, 20160296 (2016). 27335223 10.1098/rsif.2016.0296 PMC4938092

[22] V. N. Anisimov, J. A. Herbst, A. N. Abramchuk, A. V. Latanov, R. H. R. Hahnloser, A. L. Vyssotski, Reconstruction of vocal interactions in a group of small songbirds. Nat. Methods 11, 1135–1137 (2014). 25262206 10.1038/nmeth.3114

[23] J. L. Yorzinski, G. L. Patricelli, J. S. Babcock, J. M. Pearson, M. L. Platt, Through their eyes: Selective attention in peahens during courtship. J. Exp. Biol. 216, 3035–3046 (2013). 23885088 10.1242/jeb.087338 PMC4074220

[24] R. Kays, M. C. Crofoot, W. Jetz, M. Wikelski, Terrestrial animal tracking as an eye on life and planet. Science 348, aaa2478 (2015). 26068858 10.1126/science.aaa2478

[25] A. J. King, G. Fehlmann, D. Biro, A. J. Ward, I. Fürtbauer, Re-wilding collective behaviour: An ecological perspective. Trends Ecol. Evol. 33, 347–357 (2018). 29627203 10.1016/j.tree.2018.03.004

[26] W. Jetz, G. Tertitski, R. Kays, U. Mueller, M. Wikelski, Biological earth observation with animal sensors. Trends Ecol. Evol. 37, 293–298 (2022). 35263561 10.1016/j.tree.2021.11.011

[27] C. E. Beardsworth, E. Gobbens, F. van Maarseveen, B. Denissen, A. Dekinga, R. Nathan, S. Toledo, A. I. Bijleveld, Validating ATLAS: A regional-scale high-throughput tracking system. Methods Ecol. Evol. 13, 1990–2004 (2022).

[28] A. Flack, M. Nagy, W. Fiedler, I. D. Couzin, M. Wikelski, From local collective behavior to global migratory patterns in white storks. Science 360, 911–914 (2018). 29798883 10.1126/science.aap7781

[29] A. M. Wilson, T. Y. Hubel, S. D. Wilshin, J. C. Lowe, M. Lorenc, O. P. Dewhirst, H. L. A. Bartlam-Brooks, R. Diack, E. Bennitt, K. A. Golabek, Biomechanics of predator–prey arms race in lion, zebra, cheetah and impala. Nature 554, 183–188 (2018). 29364874 10.1038/nature25479

[30] M. Al-Yassary, K. Billiaert, G. S. Antonarakis, S. Kiliaridis, Evaluation of head posture using an inertial measurement unit. Sci. Rep. 11, 19911 (2021). 34620956 10.1038/s41598-021-99459-7 PMC8497508

[31] F. Kano, J. Walker, T. Sasaki, D. Biro, Head-mounted sensors reveal visual attention of free-flying homing pigeons. J. Exp. Biol. 221, jeb183475 (2018). 30190414 10.1242/jeb.183475

[32] H. Yu, J. Deng, T. Leen, G. Li, M. Klaassen, Continuous on-board behaviour classification using accelerometry: A case study with a new GPS‐3G‐bluetooth system in pacific black ducks. Methods Ecol. Evol. 13, 1429–1435 (2022).

[33] T. Eliav, S. R. Maimon, J. Aljadeff, M. Tsodyks, G. Ginosar, L. Las, N. Ulanovsky, Multiscale representation of very large environments in the hippocampus of flying bats. Science 372, eabg4020 (2021). 34045327 10.1126/science.abg4020

[34] A. Sarel, S. Palgi, D. Blum, J. Aljadeff, L. Las, N. Ulanovsky, Natural switches in behaviour rapidly modulate hippocampal coding. Nature 609, 119–127 (2022). 36002570 10.1038/s41586-022-05112-2 PMC9433324

[35] A. Weissbrod, A. Shapiro, G. Vasserman, L. Edry, M. Dayan, A. Yitzhaky, L. Hertzberg, O. Feinerman, T. Kimchi, Automated long-term tracking and social behavioural phenotyping of animal colonies within a semi-natural environment. Nat. Commun. 4, 2018 (2013). 23771126 10.1038/ncomms3018

[36] P. C. Bala, B. R. Eisenreich, S. B. M. Yoo, B. Y. Hayden, H. S. Park, J. Zimmermann, Automated markerless pose estimation in freely moving macaques with OpenMonkeyStudio. Nat. Commun. 11, 4560 (2020). 32917899 10.1038/s41467-020-18441-5 PMC7486906

[37] S. Ballesta, G. Reymond, M. Pozzobon, J.-R. Duhamel, A real-time 3D video tracking system for monitoring primate groups. J. Neurosci. Methods 234, 147–152 (2014). 24875622 10.1016/j.jneumeth.2014.05.022

[38] C. Ionescu, D. Papava, V. Olaru, C. Sminchisescu, Human3. 6m: Large scale datasets and predictive methods for 3d human sensing in natural environments. IEEE Trans. Pattern Anal. Mach. Intell. 36, 1325–1339 (2013). 10.1109/TPAMI.2013.248 26353306

[39] U. Waldmann, H. Naik, N. Máté, F. Kano, I. D. Couzin, O. Deussen, B. Goldlücke, I-MuPPET: Interactive multi-pigeon pose estimation and tracking, in *Pattern Recognition: 44th DAGM German Conference, DAGM GCPR 2022, Konstanz, Germany, September 27–30, 2022, Proceedings* (Springer, 2022), pp. 513–528.

[40] H. Naik, A. H. H. Chan, J. Yang, M. Delacoux, I. D. Couzin, F. Kano, M. Nagy, 3D-POP-An automated annotation approach to facilitate markerless 2D-3D tracking of freely moving birds with marker-based motion capture, in *Proceedings of the IEEE/CVF Conference on Computer Vision and Pattern Recognition* (IEEE Computer Society, 2023), pp. 21274–21284.

[41] C. Fichtel, M. Manser, Vocal communication in social groups. Anim. Behav. Evol. Mech., 29–54 (2010).

[42] L. F. Hughey, A. M. Hein, A. Strandburg-Peshkin, F. H. Jensen, Challenges and solutions for studying collective animal behaviour in the wild. Philos. Trans. R. Soc. B Biol. Sci. 373, 20170005 (2018). 10.1098/rstb.2017.0005 PMC5882975 29581390

[43] J. A. Klarevas-Irby, M. Wikelski, D. R. Farine, Efficient movement strategies mitigate the energetic cost of dispersal. Ecol. Lett. 24, 1432–1442 (2021). 33977638 10.1111/ele.13763

[44] S. Vistalli, T. Jäger, L. M. Aplin, S. Wild, Tits (Paridae sp.) use social information when locating and choosing nest lining material. Behav. Ecol. Sociobiol. 77, 13 (2023).

[45] A. Attanasi, A. Cavagna, L. Del Castello, I. Giardina, T. S. Grigera, A. Jelić, S. Melillo, L. Parisi, O. Pohl, E. Shen, M. Viale, Information transfer and behavioural inertia in starling flocks. Nat. Phys. 10, 615–698 (2014). 25264452 10.1038/nphys3035 PMC4173114

[46] T. Clutton-Brock, *Meerkat manor: Flower of the Kalahari* (Hachette UK, 2010).

[47] M. Nagy, Z. Ákos, D. Biro, T. Vicsek, Hierarchical group dynamics in pigeon flocks. Nature 464, 890–893 (2010). 20376149 10.1038/nature08891

[48] B. P. Hayes, W. Hodos, A. L. Holden, J. C. Low, The projection of the visual field upon the retina of the pigeon. Vision Res. 27, 31–40 (1987). 3617545 10.1016/0042-6989(87)90140-4

[49] H.-O. Nalbach, F. Wolf-Oberhollenzer, K. Kirschfeld, The pigeon’s eye viewed through an ophthalmoscopic microscope: Orientation of retinal landmarks and significance of eye movements. Vision Res. 30, 529–540 (1990). 2339507 10.1016/0042-6989(90)90065-s

[50] A. L. Vyssotski, A. N. Serkov, P. M. Itskov, G. Dell’Omo, A. V. Latanov, D. P. Wolfer, H.-P. Lipp, Miniature neurologgers for flying pigeons: Multichannel EEG and action and field potentials in combination with GPS recording. J. Neurophysiol. 95, 1263–1273 (2006). 16236777 10.1152/jn.00879.2005

[51] H.-P. Lipp, A. L. Vyssotski, D. P. Wolfer, S. Renaudineau, M. Savini, G. Tröster, G. Dell’Omo, Pigeon homing along highways and exits. Curr. Biol. 14, 1239–1249 (2004). 15268853 10.1016/j.cub.2004.07.024

[52] D. Biro, D. J. T. Sumpter, J. Meade, T. Guilford, From compromise to leadership in pigeon homing. Curr. Biol. 16, 2123–2128 (2006). 17084696 10.1016/j.cub.2006.08.087

[53] G. Dell’Ariccia, G. Dell’Omo, D. P. Wolfer, H.-P. Lipp, Flock flying improves pigeons’ homing: GPS track analysis of individual flyers versus small groups. Anim. Behav. 76, 1165–1172 (2008).

[54] M. Nagy, G. Vásárhelyi, B. Pettit, I. Roberts-Mariani, T. Vicsek, D. Biro, Context-dependent hierarchies in pigeons. Proc. Natl. Acad. Sci. U.S.A. 110, 13049–13054 (2013). 23878247 10.1073/pnas.1305552110 PMC3740899

[55] T. Sasaki, N. Masuda, R. P. Mann, D. Biro, Empirical test of the many-wrongs hypothesis reveals weighted averaging of individual routes in pigeon flocks. iScience 25, 105076 (2022). 36147962 10.1016/j.isci.2022.105076 PMC9485075

[56] J. R. Usherwood, M. Stavrou, J. C. Lowe, K. Roskilly, A. M. Wilson, Flying in a flock comes at a cost in pigeons. Nature 474, 494–497 (2011). 21697946 10.1038/nature10164 PMC3162477

[57] M. Papadopoulou, H. Hildenbrandt, D. W. E. Sankey, S. J. Portugal, C. K. Hemelrijk, Emergence of splits and collective turns in pigeon flocks under predation. R. Soc. Open Sci. 9, 211898 (2022). 35223068 10.1098/rsos.211898 PMC8864349

[58] M. F. Land, Eye movements and the control of actions in everyday life. Prog. Retin. Eye Res. 25, 296–324 (2006). 16516530 10.1016/j.preteyeres.2006.01.002

[59] S. V. Shepherd, M. L. Platt, Spontaneous social orienting and gaze following in ringtailed lemurs (Lemur catta). Anim. Cogn. 11, 13–20 (2008). 17492318 10.1007/s10071-007-0083-6

[60] F. Kano, J. Call, Cross-species variation in gaze following and conspecific preference among great apes, human infants and adults. Anim. Behav. 91, 137–150 (2014).

[61] J. R. Flanagan, R. S. Johansson, Action plans used in action observation. Nature 424, 769–771 (2003). 12917683 10.1038/nature01861

[62] F. Kano, C. Krupenye, S. Hirata, M. Tomonaga, J. Call, Great apes use self-experience to anticipate an agent’s action in a false-belief test. Proc. Natl. Acad. Sci. U.S.A. 116, 20904–20909 (2019). 31570582 10.1073/pnas.1910095116 PMC6800361

[63] E. Fernández-Juricic, J. T. Erichsen, A. Kacelnik, Visual perception and social foraging in birds. Trends Ecol. Evol. 19, 25–31 (2004). 16701222 10.1016/j.tree.2003.10.003

[64] F. Kano, T. Sasaki, D. Biro, Collective attention in navigating homing pigeons: Group size effect and individual differences. Anim. Behav. 180, 63–80 (2021).

[65] A. Itahara, F. Kano, “Corvid tracking studio”: A custom-built motion capture system to track head movements of corvids. Jpn. J. Anim. Psychol. 72, 1–16 (2022).

[66] A. Wohlschläger, R. Jäger, J. D. Delius, Head and eye movements in unrestrained pigeons (*Columba livia*). J. Comp. Psychol. 107, 313–319 (1993).

[67] F. Kano, H. Naik, G. Keskin, I. D. Couzin, M. Nagy, Head-tracking of freely-behaving pigeons in a motion-capture system reveals the selective use of visual field regions. Sci. Rep. 12, 19113 (2022). 36352049 10.1038/s41598-022-21931-9 PMC9646700

[68] S. B. Rosenthal, C. R. Twomey, A. T. Hartnett, H. S. Wu, I. D. Couzin, Revealing the hidden networks of interaction in mobile animal groups allows prediction of complex behavioral contagion. Proc. Natl. Acad. Sci. U.S.A. 112, 4690–4695 (2015). 25825752 10.1073/pnas.1420068112 PMC4403201

[69] J. R. Stowers, M. Hofbauer, R. Bastien, J. Griessner, P. Higgins, S. Farooqui, R. M. Fischer, K. Nowikovsky, W. Haubensak, I. D. Couzin, K. Tessmar-Raible, A. D. Straw, Virtual reality for freely moving animals. Nat. Methods 14, 995–1002 (2017). 28825703 10.1038/nmeth.4399 PMC6485657

[70] K. Hori, S. Watanbe, An application of the image processing system for detecting and controlling pigeon’s peck location. Behav. Brain Res. 26, 75–78 (1987). 3675837 10.1016/0166-4328(87)90018-0

[71] Vicon Tracker Software; Vicon Metrology Solutions.

[72] R. Hartley, A. Zisserman, *Multiple View Geometry in Computer Vision* (Cambridge Univ. Press, 2011).

[73] M. Bateson, L. Asher, The European starling, R. Hubrecht, J. Kirkwood, Eds., in *UFAW Handbook on the Care and Management of Laboratory Animals and Other Animals Used in Scientific Procedures, * 697-705 (Wiley-Blackwell, 8th edition, 2010).

[74] M. J. Taitt, Winter food and feeding requirements of the starling. Bird Study 20, 226–236 (1973).

[75] N. Tarcai, C. Virágh, D. Ábel, M. Nagy, P. L. Várkonyi, G. Vásárhelyi, T. Vicsek, Patterns, transitions and the role of leaders in the collective dynamics of a simple robotic flock. J. Stat. Mech. 2011, P04010 (2011).

[76] M. H. M. Menz, M. Scacco, H.-M. Bürki-Spycher, H. J. Williams, D. R. Reynolds, J. W. Chapman, M. Wikelski, Individual tracking reveals long-distance flight-path control in a nocturnally migrating moth. Science 377, 764–768 (2022). 35951704 10.1126/science.abn1663

