# Supplementary material for: SMART-BARN: Scalable multimodal arena for real-time tracking behavior of animals in large numbers
Source: Sci Adv. 2023 Sep 1;9(35):eadf8068. doi: 10.1126/sciadv.adf8068 (PMC10854427; doi:10.1126/sciadv.adf8068)
Supplement: Supplementary file 1 — Supplementary Text Figs. S1 to S15 Tables S1 and S2 Legends for movies S1 to S4 [file sciadv.adf8068_sm.pdf]

Science Advances
AAAS

Supplementary Materials for

SMART-BARN: Scalable multimodal arena for real-time tracking behavior of animals in large numbers

Máté Nagy *et al.*

Corresponding author: Máté Nagy, nagymate@hal.elte.hu; Iain D. Couzin, icouzin@ab.mpg.de

Sci. Adv. **9**, eadf8068 (2023)
DOI: 10.1126/sciadv.adf8068

The PDF file includes:

Supplementary Text
Figs. S1 to S15
Tables S1 and S2
Legends for movies S1 to S4

Other Supplementary Material for this manuscript includes the following:

Movies S1 to S4

Supplementary Text

Glossary

bio-loggers: Electronic devices used to record behavioral traits of animals using various types of sensors. The devices are attached to the body of the animal and they are used to measure physiological data such as heart rate, breathing rate, etc. or to record movement using global position (GPS) and/or inertial measurement sensors.

feedback is the return back into a system (or a machine) of part of what it produces, especially to improve what is produced.

interactive units are electronic devices or sensors that can be activated or deactivated to respond to the animals, the trigger can be programmed based on movement of the animal or audio/visual cue depending on the need of the experiment.

modalities: Modality is a term used to distinguish between different ways (or modes) of information transfer.

model organisms: A specific group of animals that are used for a wide range experiments and studies. These animals are selected due to ease of handling and maintaining in controlled environments.

modular: A modular system is made of multiple modules that are ideally operating independent of each other. Modular systems allow customization as users can select modules that they need for the experiment. Modular approach makes it easier to add and remove modules as technology progresses.

multimodal is a term used for a system having or involving several modalities.

open/closed loop: In an *open loop* system the desired output does not depend on the control action. While in the *closed loop* system the desired output depends on the control action of the system. The closed loop systems are characterized by a feedback mechanism.

pose is a term used for describing a unique position and orientation of a rigid object with respect to a specific co-ordinate system. Often described also as 6-DOF pose, which refers to six degrees of freedom each body has for movement in 3D space. Three in translation and three in rotation along the axes of the cartesian coordinate system (X, Y, Z).

position refers to locations in three dimensional metric-space as (X, Y, Z), or in two dimensional pixel-space as (x, y) in connection to images.

posture is used to describe motion or movement of an articulated object (body) that is fundamentally structured as a combination of positions and pose of joints and limbs.

real-time is referred to as the ability of a system to process the data and produce output while simultaneously collecting data (input). Real-time systems have minimal delay time and are crucial for closed-loop experiments.

scalable is a term used to describe a system that can be used at different scales depending on the need, can be made larger, for example by adding extra hardware, or deal with extra work without affecting its performance.

virtual/augmented reality: An interactive technology that enables users to visualize the real world augmented with virtual information (AR) registered to real objects in 3D or transports the user completely in a virtual world (VR).

Abbreviations

DLC: DeepLabCut (*4*)

DOF: degrees of freedom; 6-DOF refers to 3 translational and 3 rotational degrees of freedom

IR: Infrared or near-Infrared; specifying the wavelength of the light sensitivity of a camera (see also RGB) or spectrum of the light emitted by an LED.

LED: Light Emitting Diode

RECO: Remote-controlled stimulus and reward boxes (Nourizonoz et al. 2020 (*20*))

RFID: Radio Frequency Identification; a wireless system comprised of two components: tags and readers.

RGB: Red-Green-Blue; typically used for visible light spectrum when specifying the wavelength of the light sensitivity of a camera; “traditional” cameras (as compared to RGBD or IR cameras)

RGBD: Red-Green-Blue-Depth; Cameras that provide depth information linked to each pixel in the view camera. The technology is also known as 2.5D.

TOAD: time of arrival differences; a method used for calculating location of sound sources

Detailed comparison to other systems and studies

Nourizonoz et al. 2020 (*20*)

Subjects/Species: mice, mouse lemur
Focus: Closed loop experiments with multiple sensors and 3D position, 2D posture tracking.
Modality/Sensors:

Infrared Camera: 3D Position tracking with active markers for animals n >1 and passive for n = 1.
RGB Camera: Gimbal mounted close focused camera for offline behavior using deep learning.
Biologger/On body sensor: Neural recording
Stimulus: RECO - Box Audio / Visual stimulus delivery
Performance:
3D Triangulation: 785 - 580 Hz (for 1 to 3 targets). Triangulation speed will decrease with an increase in the number of individuals.
2D Posture: 93 Hz
Scalability:
Claims that the system is scalable to different sizes. 1 to 100 cubic meters.
Case Study:
1. Foraging condition with 3D Tracking + RECO + Lighting change
2. Fully automated: 3D Tracking + RECO + RGB- Posture
3. Location based optogenetic: 3D tracking + 2D Tracking
4. Brain recording: Body sensor + 3D position

Weissbrod et al. 2018 (*35*)

Subjects/Species: mice (up to 10 mice)
Focus: Individual and group behavior with identity and 2D position tracking, Open loop
Modality/Sensor:
RFID implanted bio-logger - For identity (synced to video) and position +
39 Antenna. Video - 2D refined position tracking, speed, direction (n = 1) at 759 × 596 resolution.
Scalability:
Promoted for use as setup for other experiments. Used arena: 119 × 119 × 80 cm
Performance:
RFID: +/- 8-11 cm
RFID + Video: +/- 0.5 cm with 30 ms temporal precision (video is 30 fps)
Identity swap: maintains 94% with 10 mice. Drops with increase in individuals. Additional software used to reduce swapping.
Optimization: how many antennas are enough for optimal performance?
0.0018 antennas per cm^2. 120×120 cm requires a minimum 23 antennas but they use 39 for the same area.
Case studies:
Phenotyping of mouse groups: 8 days
Individual locomotion patterns within group
Social interaction between pairs
Social hierarchy formation and stability in mouse colonies

OpenMonkeyStudio, Bala et al. 2020 (*36*)

Subjects/Species: Rhesus macaques
Focus: Markerless 3D posture tracking, individual identification (n = 2);

setup is promoted with idea of markeless behavior understanding with 3D posture and identity
Modality/Sensors: RGB Camera (n = 60)
Scalability: 2.45 × 2.45 × 2.75 meters
Authors do suggest that a similar setup is possible to scale to other species.
Performance:
2 individuals, Offline operation on posture
Case studies: Comparison with other marker-based video motion capture
Quantitative evaluation:
Head trajectory on 30 min sequence reports reconstruct accuracy with a median error of 6.76 cm, a mean error of 7.14 cm and a standard deviation: 2.34 cm.
Justified multi-view augmentation leads to better prediction
Identification of meaningful actions: 3D vs 2D sequence. Used a 30 min sequence to show actions like walking, standing, climbing.
Climbing upside down are better registered with 3D than with 2D.
Social context: 2 individuals tracked while interacting.
Dataset: Using 3D reconstruction to create large annotations with smart modifications

Sarel et al. (Ulanovsky) 2022 (*34*)

Subjects/Species: Egyptian fruit bats (n = 4, but an experiment have only 2 bats at a time or solo flights)
Focus: Open loop
Neural encoding with spatial movement and echolocation
Flying, collision avoidance
Modality/Sensors: 2D Position tracking in cave: Wireless RFID (bio-logger), active tags (6.6 g, Bespoon) with 14 Antenna RFID receivers, Neural recording (synced to RFID)
Audio loggers (bio-logger)
*used simultaneously and independently with different hardware
Scalability:
135 meter long tunnel × 2.3 m × 2.3 m
Setup is not promoted for replication
Performance:
Longitude - Latitude error: 9 cm (Height estimation is poor and not used)
Triangulation rate: 12.8 to 16 Hz (Up-sampled to 100 Hz for analysis)
Acoustic recording: 100 kHz sample rate, 4-40 kHz filtering
Case studies:
Experiments with bats flying solo or in group, recording position 2D, acoustic vocalization and neural activity.

Chaumont et al. 2019 (*8*)

Subjects/Species: Mice
Focus: Real-time (Closed loop)

Automated Individual identification with real-time tracking for individual and collective behavior in mice for long term experiments
Modality/Sensor: RFID - Identity and tracking validation
RGBD - Depth sensor for 2.5D tracking (shape + posture)
Scalability:
Promoted and used in multiple locations after being proposed
Reproducible hardware, calibration protocol and data acquisition explained in detail
Provide data visualization tool, online data sharing support
Performance:
The system can operate for several hours to days.
Case studies:
Reproducible hardware, automatic phenotyping, on-the-fly monitoring tracking quality + behavior.

PRIME3D: Ballesta et al. 2014 (*37*)

Subjects/Species: Primate
Focus: 3D position tracking (x,y,z) of multiple individuals with identity for social behavior understanding
Modality/Sensor: RGBD - Multi-camera with colored collars
Scalability:
1.5 m × 2 m × 2 m
Number of individuals can be increased based on ability to make new collar tags
Performance:
Real-time
3D Position with identity for 4 individuals at 15 Hz
Spatial resolution: 1 cm
Case studies:
Behavior separation is done based on the tracking

DANNCE, Dunn et al. 2021 (*9*)

Subjects/Species: rats, mice, marmoset, chickadees
Focus: 3D Posture, Method to computer 3D posture of animals without markers and further use to quantify behavior patterns. Works for a single individual.
Modality/Sensor:
Motion capture system + 3-6 RGB cameras. The setup is custom-made and dimensions of setup are not available.
Scalability:
No comments on scalability of setup, but code is provided so that method can be used with other species given hardware criterion is met.
Performance:

3D posture prediction from single and multi-view, Behavior categorization compared with human annotation.
In rats, 6-camera DANNCE predictions relative to hand-labeled points (8.4±4.6 mm) was close to the error
between the human labelers themselves (8.0±4.8 mm), 3 cameras were used (DANNCE, 9.4±5.9 mm). Further work on this approach with "PAIR-R24M Dataset" reports error of 6.4 mm on rats (*10*).
Case Studies:
Made large datasets with different animals and demonstrate the multi-view or single view 3D reconstruction of posture using the millions of training data created from mo-cap or manual annotations. The method uses volumetric information and temporal information to predict better posture then multi-view
Triangulation using DeepLabCut(*5*). Mo-cap data is only available with rats, birds and marmoset and bird data is human-annotated.

Anisimov et al. 2014 (*22*)

Subjects/Species: Zebra finch
Focus:
Recording and analyzing individual vocalizations in free ranging birds.
Sounds are recorded with a microphone and acceleration is used to identify the individual that makes the sound.
Relative distances are measured with sound amplitude and recording from other microphones.
Modality/Sensor:
Audio - Acceleration loggers (13-17 g), Offline experiments
Scalability:
The method is demonstrated with n = 4 subjects.
Spatial locations and sensitivity of microphone may be crucial in increase to large area, Small setup (< 0.5 × 0.5m) but could not find exact dimension.
Performance:
System is able to associate individuals with calls in temporal data.
Case studies:
Different experiments with 1 or 4 birds show that the system can be used to identify calls from individuals and then study behavior based on call sequences.

Extended Materials and Methods

Experimental area

The physical environment dimensions are 14.68 × 6.62 × 2.2 - 3.83 (length × width × height) in meters. The adjustable 'ceiling' allows manipulating volume of the experimental area from 231 m^3 to 372 m^3. The tracking area can be enclosed with different types of barriers such as a removable polypropylene (PP) net (10 mm mesh; 0.8 mm strand width) impregnated with flame retardant (made by Heberle Altusried) to keep animals within the area, and removable white

Tyvek© (DuPont de Nemours, Inc., USA) panels on all sides to restrict visibility outside the net. This environment was designed for highest standards to be able to work with a wide variety of wild and captive animals (fulfilling animal ethics, laws and regulation standards, for example using hormone-free paints, providing durable but collision-safe netting, non-flickering lights, etc.). The experimental area also supports high precision light control. The space is designed to block any outside light (visible, infrared, ultraviolet, etc.) and the overhead light system (Zumtobel Craft L LED 32K-827-865 PCWB LDE WH DT6) can be programmed (using a Zumtobel Litecom programmable unit) to emit light in specific spectra (e.g., natural light), levels (e.g., dawn), and scheduled times (e.g., following current daylight timing). This allows for full manipulation of the light in the experimental environment.

Motion capture system: 3D tracking with Infrared cameras

We deployed the commercially available Vicon© motion capture (mo-cap) system for marker based tracking solution. Our setup in the SMART-BARN consists of 30 Infrared (IR) cameras: 26 Vero v2.2, (2048 × 1088 pixels resolution), 4 Vantage V5 (2432 × 2048 pixels resolution); providing data at high frame rate (between 50 and 300 Hz; see table S1) to capture motion of fast moving objects in three dimensional spaces. The system is ideal for animal tracking applications because Infrared cameras operate at high frequencies and provide results in real-time (typically the latency is less than 10 ms when using through networking, but Vicon Tracker can process and provide data as low as 1.5 ms (*71*)). In the following text we will highlight operational basics and specification of the equipment used in our setup.

Camera position and marker selection

The mo-cap (Infrared; IR) cameras are positioned to achieve maximum coverage in the entire tracking volume (to be calculated with optimized tracking between 0 and around 2.5 m from the ground), but this can readily be modified based on the need of the experiment. Mo-cap systems work on the principle of stereo triangulation and therefore each camera must be positioned such that areas of interest are visible in the field of view of at least two cameras. The system operates with two types of markers: (*i*) passive markers and (*ii*) active markers.

Passive markers are spherical in shape and the surface is coated with retro-reflective material that reflects infrared light homogeneously (6.4 mm in diameter by OptiTrack© company were used for projects needed highest accuracy of small markers, and 9.5 mm diameter markers by Vicon were used as well). Each camera is surrounded by a ring of IR-LEDs (strobes; 850nm wavelength) that illuminate the scene with IR light. This method is efficient because markers appear as bright circular spots in the images that are easy to detect with minimal processing and thus the system operates at higher framerates. Triangulation is achieved in real-time in a global coordinate system.

Active markers are made with IR light source (Vicon Active Wand has 5 pairs of Near Infra Red / Infra Red and Visible Red LEDs; the latter for RGB cameras, see later) and do not require IR strobes of the cameras to be turned on. Active markers require their own power supply and can be configured to emit lights at different frequencies or intervals to create smart and unique markers for identity tracking.

The accuracy of the system is in the range of sub-mm (*71*). It may vary based on the size of the markers used and their distances from the camera. Researchers can select markers of various sizes (see Table S1) based on the need of the experiment i.e. tracking volume, size of animal,

tracking strategy (point, pattern or skeleton). Smaller markers and patterns designed with such markers are compact in design which is ideal for tracking smaller animals because of weight considerations. Overall tracking performance is a function of multiple factors such as position of cameras, overlapping areas, size of marker, movement of animals etc.

Tracking features

Marker tracking is used in three ways: point tracking, pattern tracking and skeleton tracking (see also Table S2). Users have to use one of the three features to get desired tracking results. In this subsection, we will briefly go through the advantages and disadvantages of using each and their operational limitations.

Point tracking function allows 3D position tracking of a single marker. The position of all markers are recorded and stored, including the raw 2D positions on each camera, so 3D positions can be later recalculated using different calibration data. The live 3D positions can be sent via broadcast to connected devices within approx. 10ms delay for online/real-time use. Typically, mo-cap processes data at 1.5 ms and up to 500 frames per second (*71*) and additional delays are introduced when extra data processing is done at client side for closed-loop experiments. Mo-cap system does offer post-processing options to connect tracking results of the markers over time to create trajectories (offline). The manufacturers do not reveal the algorithms that are used to create trajectories. It is possible that the same marker is assigned a different identity in the same session if tracking is lost due to line of sight or occlusion. It is possible to manually join trajectories through the software interface.

For experiments with animals, it is helpful to create customized scripts to assign trajectories using experimental constraints e.g., number of markers present in the scene and direction of motion. This method provides the means to track a large number of entities but does not resolve identity. Although individual marker identities are kept for continuous tracking segments. A single marker is lightweight, easy to attach and ideal for experiments with very small animals (insects). This approach is useful for experiments where identity tracking is not relevant e.g. tracking movement of a single animal. The markers are also useful for measuring position of static features in the scene that do not need identity specific tracking e.g. perch corners, feeder boxes. It is possible to resolve identity with point tracking if active markers are designed and used as demonstrated by Nourizonoz et al. 2020 (*20*). However, active markers are bulky to design because power requirements for the IR-LED.

Pattern tracking an extension of point tracking feature. Four or more markers form a specific geometric configuration (3D or planar) in terms of distances and angles with respect to each other. If marker positions do not change in relation to each other during the experiment then the geometric relationships are maintained and each constellation can be identified uniquely as a pattern for the entire duration of the experiment. We refer to this feature as pattern tracking and the results of pattern tracking are provided in terms of 6-DOF (degrees of freedom) pose. 6-DOF pose is the position (X, Y, Z in mm) and orientation (RX, RY, RZ in radians; or RW, RX, RY, RZ as quaternions) the marker pattern w.r.t global coordinate system.

In behavior experiments, identity tracking is essential for experiments that focus on larger groups. Pattern tracking maintains identification and therefore provides long term 3D trajectory of the animal even if tracking is lost at times due to occlusion or animal being outside coverage area. 6-DOF tracking has an additional advantage of tracking the precise orientation of all

objects in same coordinate system. This is helpful when orientation of the pattern can yield additional information such as which direction the animal is facing or looking at (see case study B for head tracking). In terms of pattern design, it is better to have 3D patterns over 2D patterns to avoid symmetric patterns, where the 3D orientation is ambiguous. If patterns are too similar, their identification algorithm makes mistakes and the errors have to be corrected in post processing. It is ideal to have a compact 3D pattern with markers of right size to obtain seamless tracking experience.

Skeleton tracking feature is built upon the idea of combining point and pattern tracking to track posture of an articulated object with a fixed number of joints and limbs. Each limb in the articulated body is attached to a special pattern and single markers are attached closer to the joints. The complete skeleton is defined as a skeleton template in the software by defining joints and limbs. The template can be designed for any articulated body provided minimum conditions of marker attachments are satisfied. Typically, one of the joints is defined as the root joint and pose (position, orientation) of each joint is provided w.r.t the root joint along with the corresponding angle between the joints. The pose of the root joint is provided in the global coordinate system.

For behavior experiments to be performed in the SMART-BARN, the setup is ideal to measure the full skeleton of large animals (e.g., cats, dogs, humans). However, we also show that the same method can be used with smaller animals, such as pigeons (see case study B). In this case, we simplify the articulation to a single ball joint.

Output

The measurements of the mo-cap system can be accessed in two ways (*i*) online (real-time) via streaming or Broadcasting (*ii*) offline via storage files. The main difference is that online mode provides access to data at the same time as it is being captured whereas offline mode stores the data for use it in the future. In offline mode, the data is accessed through data files that are created to store the data captured during the experiment.

Online mode provides direct access to the 3D data stream in real-time over wired (ethernet) or wireless network connection. The data is mostly provided for the basic tracking features i.e. point tracking (3D point position in mm) and pattern tracking (6-DOF pose). Identification of the patterns are also resolved in the stream. Users have to write customized scripts to read the required data from the stream using the SDK provided by the manufacturer (Vicon in this case). The online modes are used during closed-loop experiments, where tracking results are processed via customized scripts (written in Python; available at https://zenodo.org/record/7890292) and used in the experiment to trigger devices or provide stimuli based on movement of the animal (see case study C). Users can also transmit the data to other wireless devices like a small sensor, robot or computing using (arduino unit).

Offline mode is accessing the recorded data using customized scripts or the software provided by the manufacturer. Mo-cap system stores information of all recorded sessions (3D positions, calibration etc.) in a proprietary format. The user can access to the data via software interface, visualize the tracking results and export it to some predefined formats e.g. *.csv. Users can choose different features from the software to do post processing operations before exporting the data e.g. filtering, trajectory correction, identity assignment, etc. The software also shows 3D tracking data mapped on to the video footage taken by the synchronized RGB cameras. Users

can write customized packages to post-process the data in a specific manner using different scripting languages.

Video recording (RGB)

Our system includes 6 video cameras (Vicon Vue) that are temporally synchronized and calibrated with the motion capture system. The cameras can be mounted in desired configurations to get video recording of the experimental arena. 3D marker tracking results are visualized in video images through proprietary software (Vicon Nexus) provided by the manufacturer. It should be noted that, it is possible to buy cameras from other manufacturers (customized or commercial) and attach them to the mo-cap system using hardware triggers (explained below). We have chosen cameras provided by the same manufacturer to simplify the process of calibration and synchronization. The software only allows mapping 3D data over video footage within the scope of the software. We have developed a customized software to replicate the same process (available at https://zenodo.org/record/7890292).). This allows us to create datasets for computer vision applications such as single or multiple bird tracking in 2D (*39*) and 3D (*40*). Figure S14 shows results of some off-the-shelf markerless tracking methods that are directly applied to the dataset created using SMART-BARN setup. In the existing system, video data stream is not accessible in the online mode due to hardware limitations specific to the manufacturer of mo-cap. In future, it is possible to add additional cameras that operate with hardware triggers and provide access to video data in online mode.

Microphone array

For acoustic recording, the SMART-BARN uses a custom-built system consisting of 30 microphones (Knowles FG-23329) fixed to the ceiling of the tracking area. The microphones have a broadband frequency response ranging from 100 Hz to 130 kHz and are able to record both audible and ultrasound signals; Figure 1E). Vocalizations and non-vocalization sounds are amplified using custom-made amplifiers (produced by the Workshop at the University of Konstanz from commercially available parts) and analog-digital converted (ADC) using four National Instruments (NI) ADC cards (NI USB-6356). The four ADC cards were synchronized with each other using a synchronization pulse produced by a master ADC card and the three remaining cards follow this as slaves. The sample rate can be adjusted to the species of interest, for example, for birds a sample rate of 100 kHz was chosen, for ultrasonic calibration and recordings of bats 300 kHz are used. Long-term continuous recordings achieved by storing all audio data (MALTA-Software by ‘CAE Software and Systems’). This allows for standard bioacoustic processing and analysis of all sounds (vocalizations, strigulation, mechanical noises, playback of recordings, etc.) as well as acoustic localization. Acoustic localization is performed in post processing using custom-written Python routines (codes available for future users open source at https://zenodo.org/record/7890239) and allows to determine the position of a sound source in 3D based on the time of arrival differences (TOADs) of a signal at the 30 microphones.

Acoustic tracking

Acoustic tracking in our setup works by triangulating a sound source. The position of a sound source (both animals and sound emitting devices) is computed by measuring the time of arrival differences (TOADs) between the microphones. After bandpass-filtering the recordings, an automated detector running on one channel detects acoustic events (or an expert can identify

events manually), and the corresponding recordings on the other 29 channels are selected accordingly as detailed later. Due to the strong signal deterioration over distance, especially of high frequencies, usually a subset of channels is automatically chosen for localization. The TOADs of the signal between those channels is measured by cross-correlation. Given the large number of receivers, resulting in an over-determined array, a relative localization error can be computed by iteratively removing receivers from the analysis. The localization accuracy is in the range of tens of centimeters (for both high and low frequencies) which typically allows identification of the individual/object that produced the sound. Sounds (timing, location, and if applicable directionality) can be merged with the data of the motion capture system in order to have a time-synchronized common global coordinate system.

We used the microphone array (Figure 1E) to record all vocalizations during the four days (a new recording file was created every 5 seconds). After experiments were completed, we used custom-written automated programs (coded in Python; available at https://zenodo.org/record/7890239) to find the time of loudest sound in each 5 second recording (sound of interest) and the microphone with the loudest recording of each sound (closest microphone). Then we calculated the time of delay (time a sound takes to hit every microphone that detects it after hitting the closest one) using cross-correlation.

Finally, using an iterative process that predicts the time and the XYZ position of the sound and compares it with the time delay for each microphone, we determined the most likely position as the one where the actual and predicted time delays had the best fit (above a specific threshold). Using the estimated timing and position, we calculated which microphone/channel has the highest squared differences, and excluded that microphone from the optimization. To accept an estimated location, two other criteria were used: (*i*) at least data of 11 microphones (lower threshold) were needed to be used; (*ii*) for which the mean remaining least squares per microphone needed to be less than $5mm^2$ (as an upper threshold).

We assigned calls to regions simply determining where in space the calls were (i.e., perching zone, foraging zone, flying zone; Figure 3C), and assigned calls to individual birds using two criteria: (*i*) the closest individual was within 30 cm, and (*ii*) the next closest individual was a minimum of 50 percent farther from the sound than the closest individual.

Custom software development

We have developed multiple scripts (specific computer code) to process the data provided by various systems used in the SMART-BARN. All of the implementations are in open git repositories and all have sufficient documentation. This text only serves as an introduction to the scripts for the readers to get an idea about the different ways of using such a system. Users can use the code (scripts) to reproduce the results or adapt the methods for other customized applications.

Data fusion with video images

The mo-cap software provides options to visualize the 3D tracking data mapped onto the video images. However, most features are restrictive because they are available only with exclusive use of the software. For example, exporting 2D positions (*x*, y coordinates) of markers in the video images is not possible with the mo-cap software. We developed customized scripts to read the 3D data exported from mo-cap software (in different formats). The scripts map 3D data to the video footages using the calibration information provided by the mo-cap system. The scripts

provided are examples and users can develop more applications using the concepts demonstrated in the scripts.

We have developed a customized software to read the position of the markers exported from the Vicon software. The exported files are able to read the standard file format specific to Vicon. Additionally, we also provide a method to read .c3d files, that are general purpose output of the motion capture systems (independent of manufacturer). The software is able to do these basic functionalities: (*i*) Point manipulation: to convert 3D position of markers from global coordinate system to local coordinate system of any camera using calibration; (*ii*) Image projection: to project 3D position of markers to image using intrinsic calibration; (*iii*) Data visualization: to draw features are provided to support the example process.

Example application: Automated dataset for machine learning

The scripts we developed to visualize data on video images for computer vision tasks that are useful for more than just visualization (see computer vision module in Figure S5, and Figure S14). We provide one example of how to use the 3D tracking data (position and pose) from the experiments to mark features on or around the animals e.g., bounding boxes. These virtual markings can be directly used as annotations for computer vision problems such as object detection algorithms. As the first step, we provide scripts to read 3D marker positions and project them onto the image space. If the animal has a pattern attached to it then we can make a sufficient estimate about the position and orientation of the bird in 3D space as a vector. Then we create a 3D bounding box around the vector with a certain assumption of the size of the animal. This box can be easily projected onto the image space and can provide annotation for state of the art object detection algorithm. The setup can generate automatically annotated datasets with thousands of images in a reliable and robust manner with very accuracy and minimal manual effort. It is also possible to obtain complex annotations for computer vision problems focused on extraction of 2D or 3D postures or reconstruction of birds from images. Such datasets are essential for development of marker-less motion capture methods for animals. In future, marker-less tracking may replace the marker based tracking or enhance the performance in cases where tracking is lost due to occlusion of markers (*40*).

Supplementary Figures

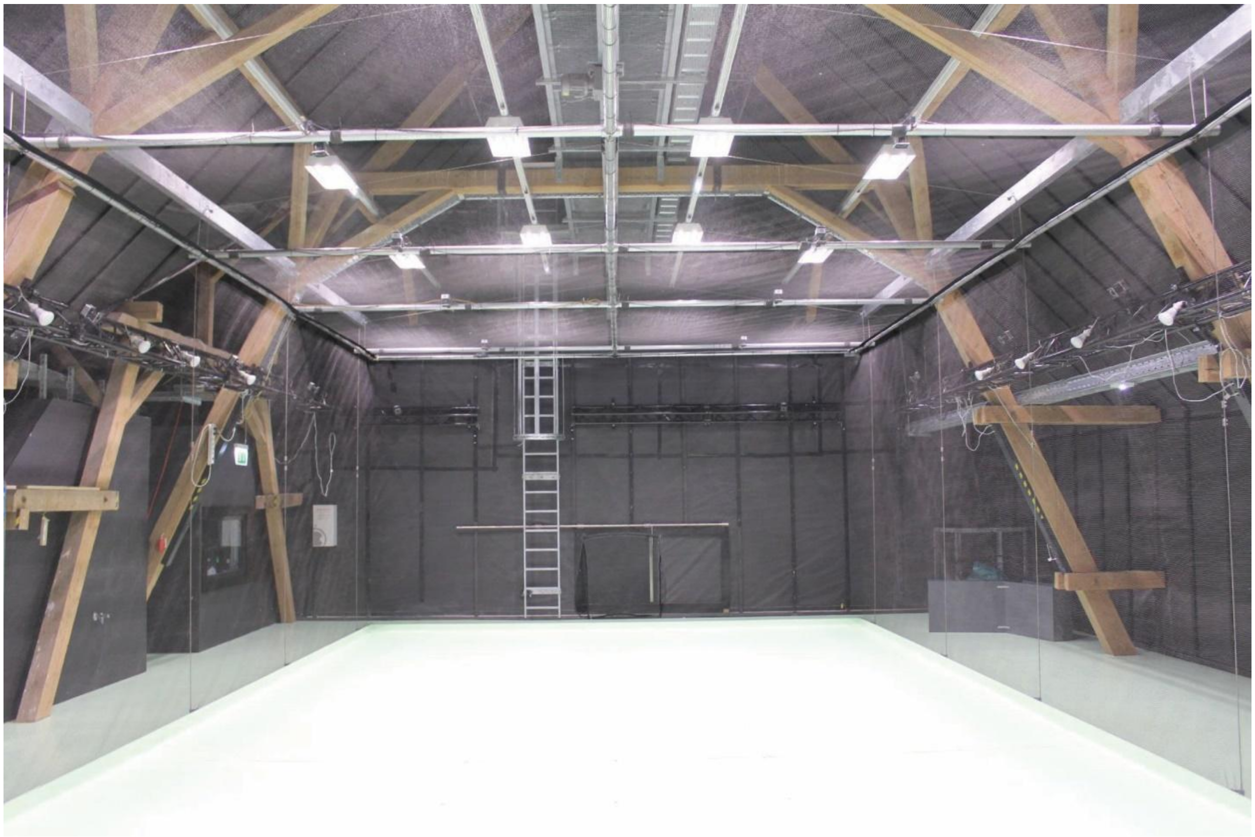

Fig. S1. Photo from inside the experimental volume in the SMART-BARN.

The photo shows the view of the empty experimental volume in-between setting up or running experiments. The photo was taken from the main entrance double door area (Figure 1C of the main manuscript; internal curtain-door not visible). Ladder to access the catwalk (depicted also on Figure 1B) and ceiling of the experimental volume visible on the back wall. Small sliding door allowing access from the experimental volume to the outside visible to the right of the ladder; net tunnel (Figure 1C) from experimental volume to external sliding door is located at the right corner, behind the Y-maze that was used in case study C.

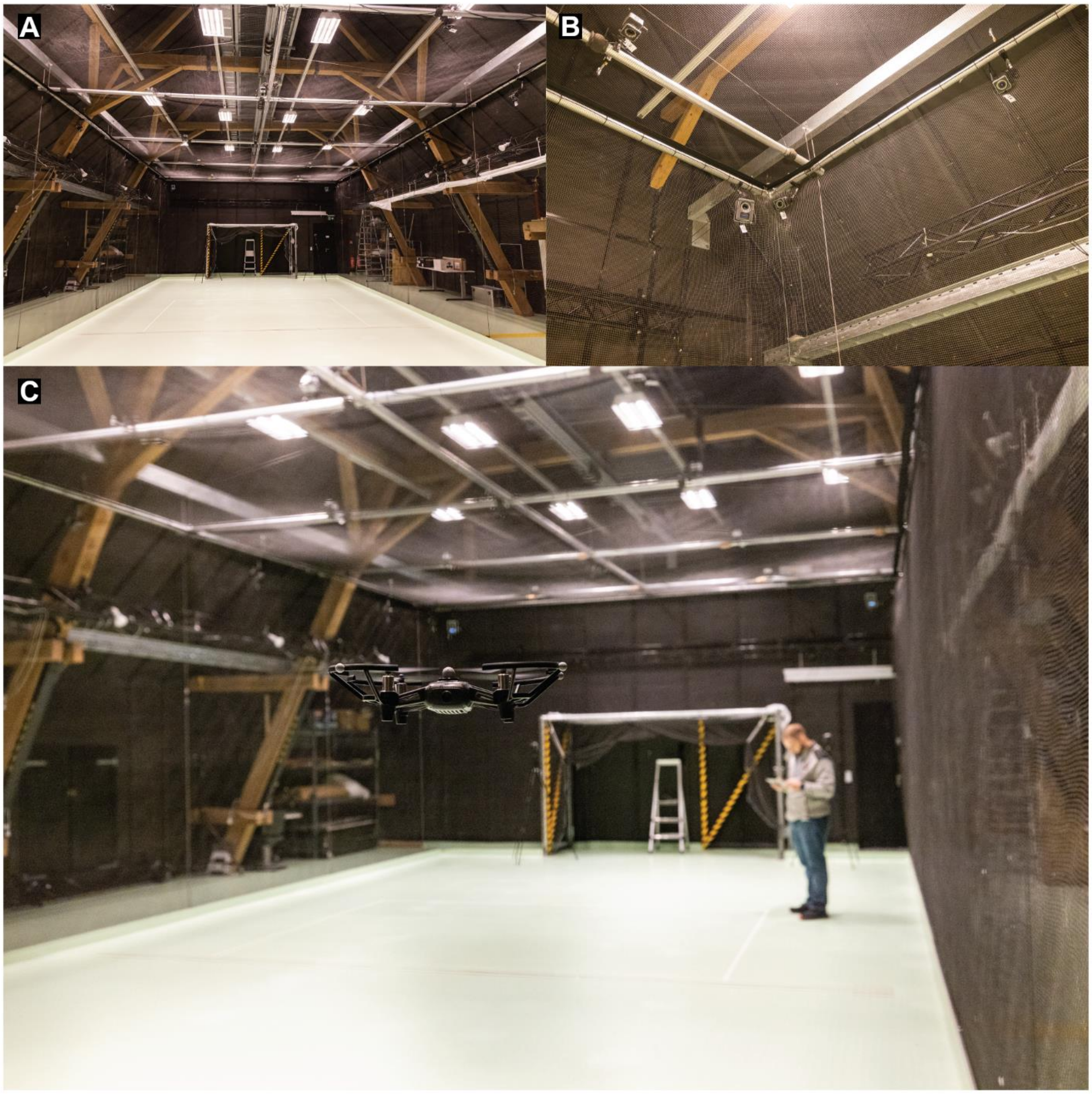

Fig. S2. Photos of the SMART-BARN.

Additional photos taken of the SMART-BARN to further illustrate the setup (photos by Christian Zeigler). (**A**) View from opposite to the main entrance with the double door (visible as the lower metal frame) Figure S1. (**B**) Close-up view of the net and the motion capture cameras in the upper corner of the volume. (**C**) A hovering quadcopter drone is shown in the foreground, with mo-cap tracking markers (gray spheres on top and edges of the quadcopter), while an experimenter controlling the copter is visible in the background. The double door entry system (an important feature that allows researchers and staff to enter without a risk of an animal escape) can be seen behind the experimenter. The internal net-door is in the open position (seen pulled into the corners) while one external swinging door is locked into the open position on the back wall (orange vertical and diagonal beams visible to the right of the ladder) and the other is locked in the closed position (vertical and diagonal beams visible to the left of the ladder).

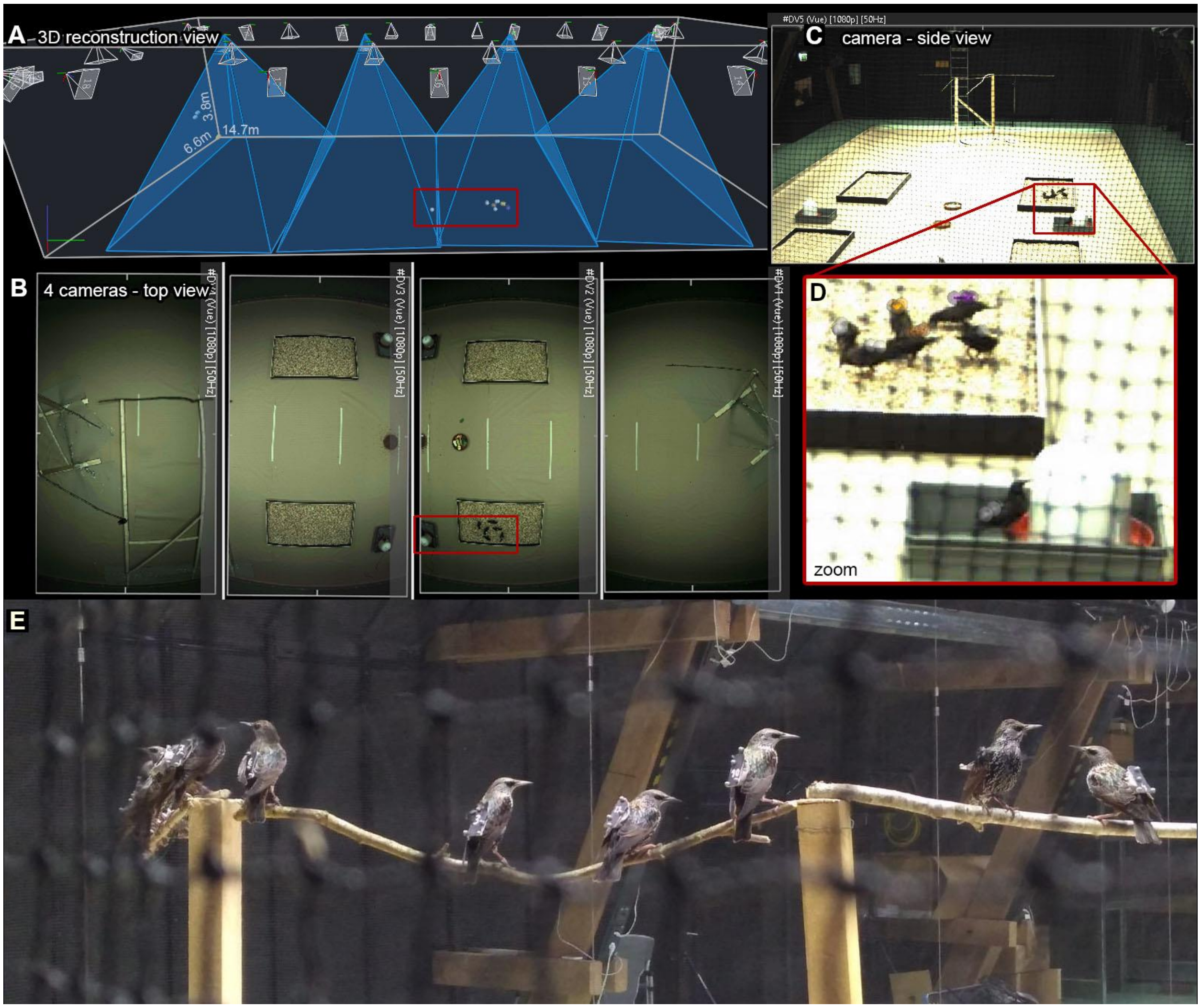

Fig. S3. Illustration of an experiment in the SMART-BARN using starlings.

Illustration shows the motion capture and video camera setup during an experiment using starlings (similar to, but different from case study A). (**A**) The motion capture system (Vicon) consists of 30 infrared (IR) sensitive cameras with additional 6 high-definition visible light cameras as shown on a 3D reconstruction view of the full volume surrounded by a net. Blue pyramids indicate the field of view of the 4 downward-facing video cameras, and small gray dots show the markers detected by the IR cameras. (**B**) View from the downward-facing cameras that can be stitched together to record behavior in the entire volume. (**C**) View from a side-facing camera placed outside the net. The same group of birds is highlighted with the red rectangle on all panels. (**D**) Enlarged view from panel **C**. Semi-transparent circles show the position of the detected markers back-projected and overlaid on the image from a side-facing video camera. (**E**) Photo of starlings perching on the branches.

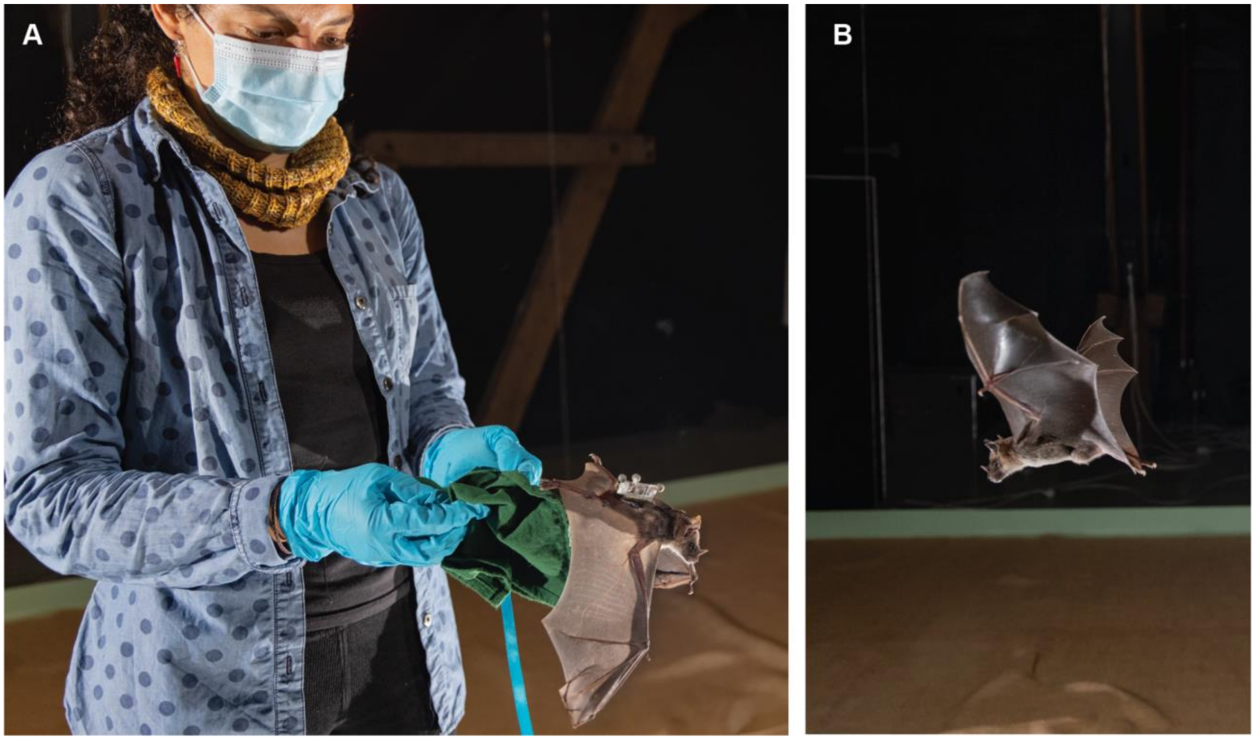

Fig. S4. Photos of an experiment in the SMART-BARN using bats. Photos (by Christian Zeigler) illustrate an experiment using bats (similar to, but different from Case example E, here the wings are not marked). (**A**) An experimenter releases a bat which is equipped with onboard sensors and a marker pattern for mo-cap tracking. (**B**) Free flying bat with markers attached to its body.

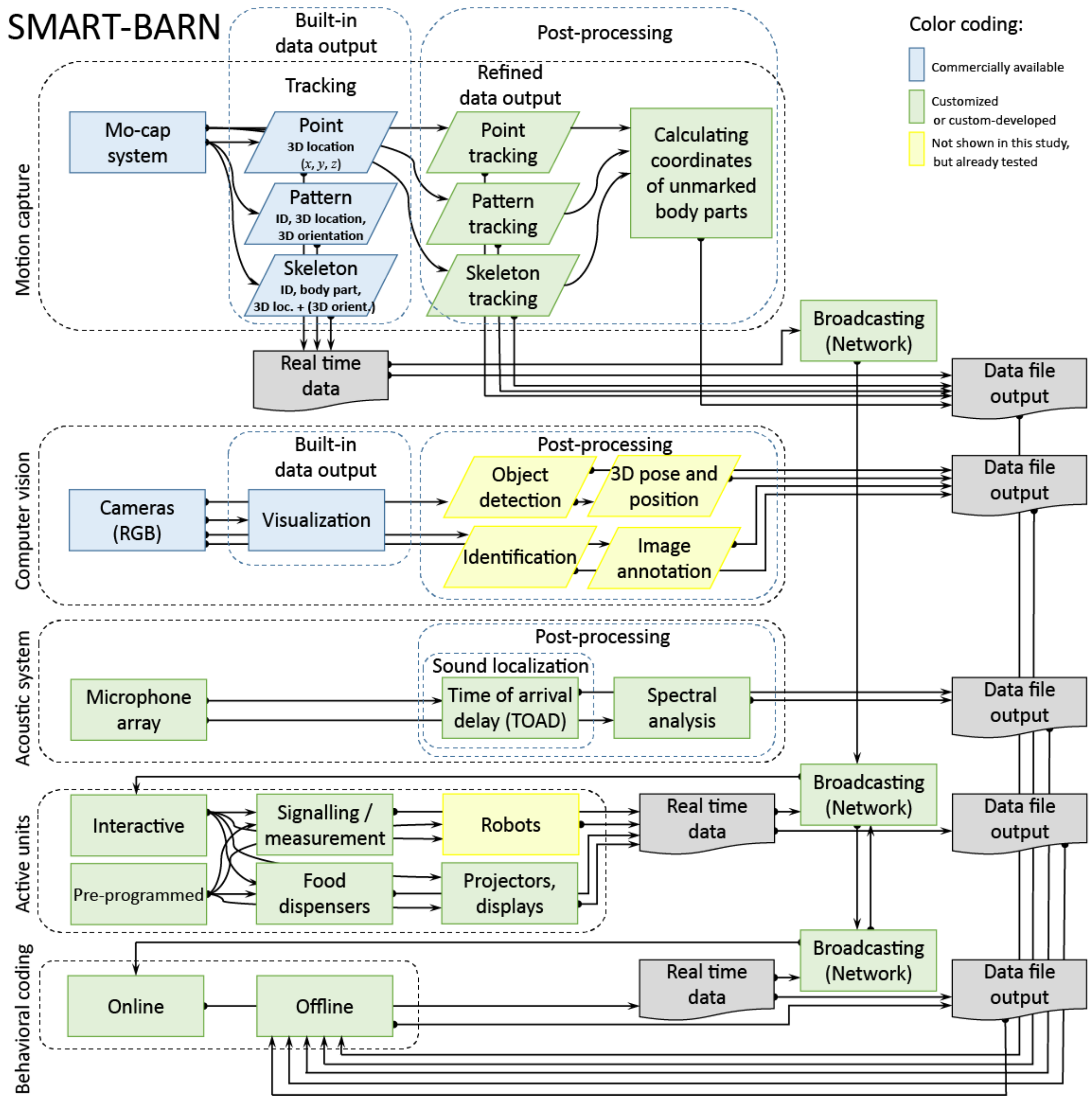

Fig. S5. Schematic illustration of the components and their connections in the SMART-BARN. The modular main components are marked with dashed lines. Color coding shows features that were available with the hardware commercially purchased (blue), needed to be customized for animals or custom-developed by us (green), or those that are not shown in the main paper (although already were tested; yellow). Broadcast network module manages communication of data in real time between the different components as short message data format via standard communication protocols (UDP; user datagram protocol). See Fig. S6-11 for the components used in the case studies and examples. For the component not shown in the main paper, see Fig. S2A and S14.

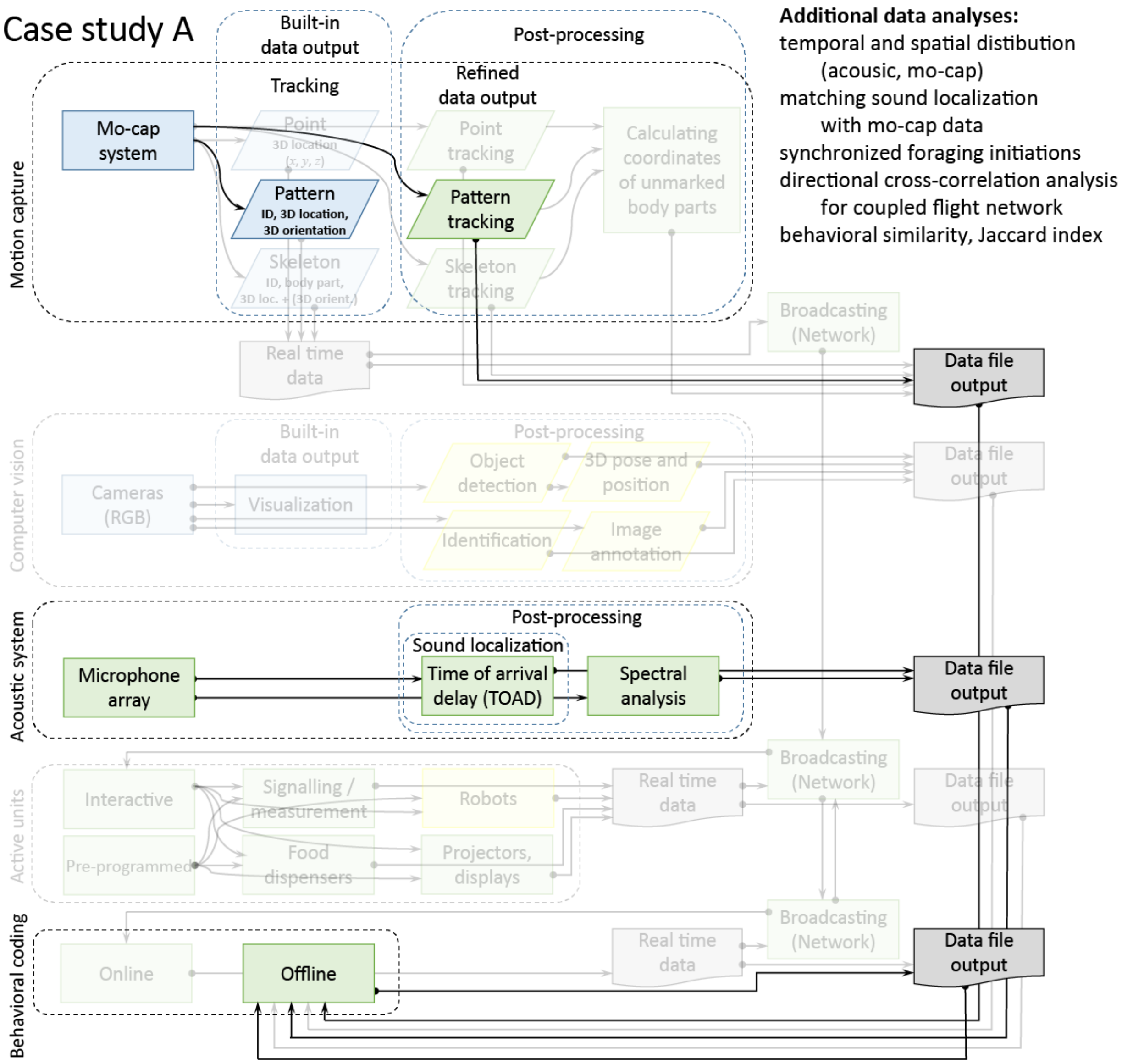

Fig. S6. Components and their connections used in Case study A. For detailed description see Fig. S5. Additional analyses listed on the top right. Note that for matching the 3D localized sound, we compared that to the location of the closed marker pattern (on the back of the bird). This introduces a specific error (in a range of 5-8cm), which results that during perching when birds are typically in each other's proximity, the calls do not get assigned to any individual (or with much less probability, they get assigned to a wrong individual). This error could be reduced by calculating an estimated position of the head (beak) of the bird using the 3D vectorial information of the backpack, as shown in Case study B, where the location of unmarked body parts (e.g. the eyes) are estimated based on the marker on the top of the head.

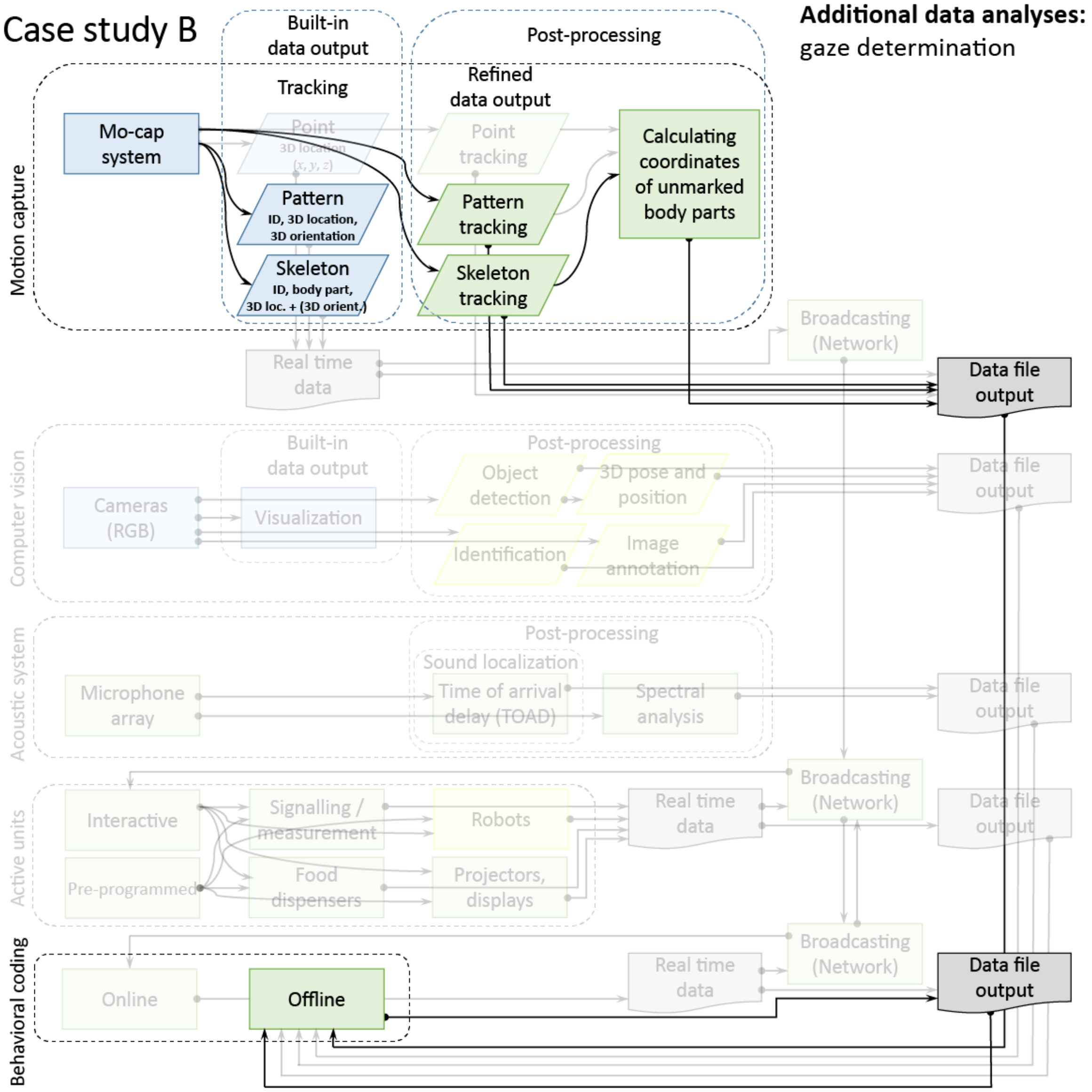

Fig. S7. Components and their connections used in Case study B. For detailed description see Fig. S5. Additional analyses listed on the top right.

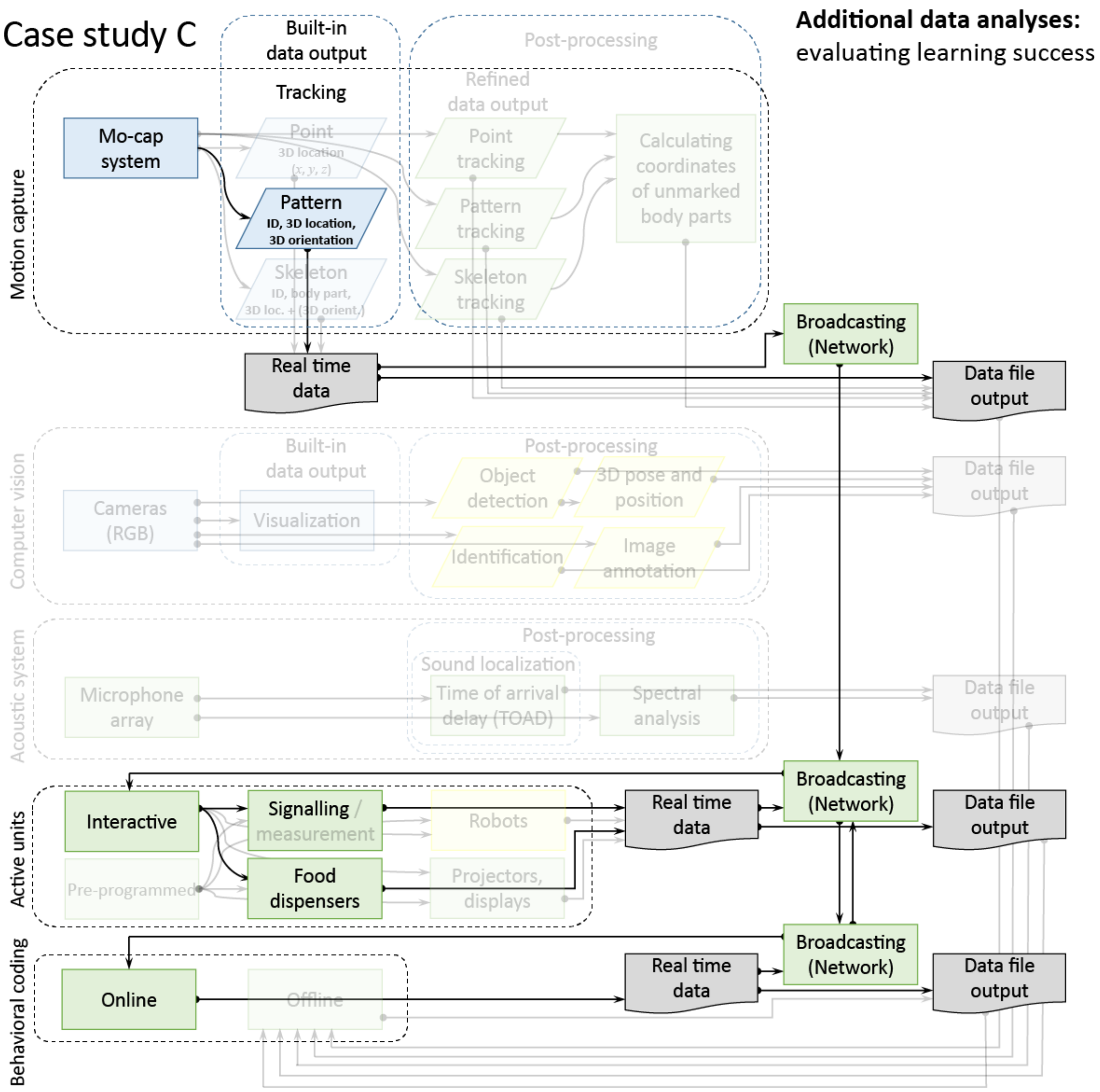

Fig. S8. Components and their connections used in Case study C. For detailed description see Fig. S5. Additional analyses listed on the top right.

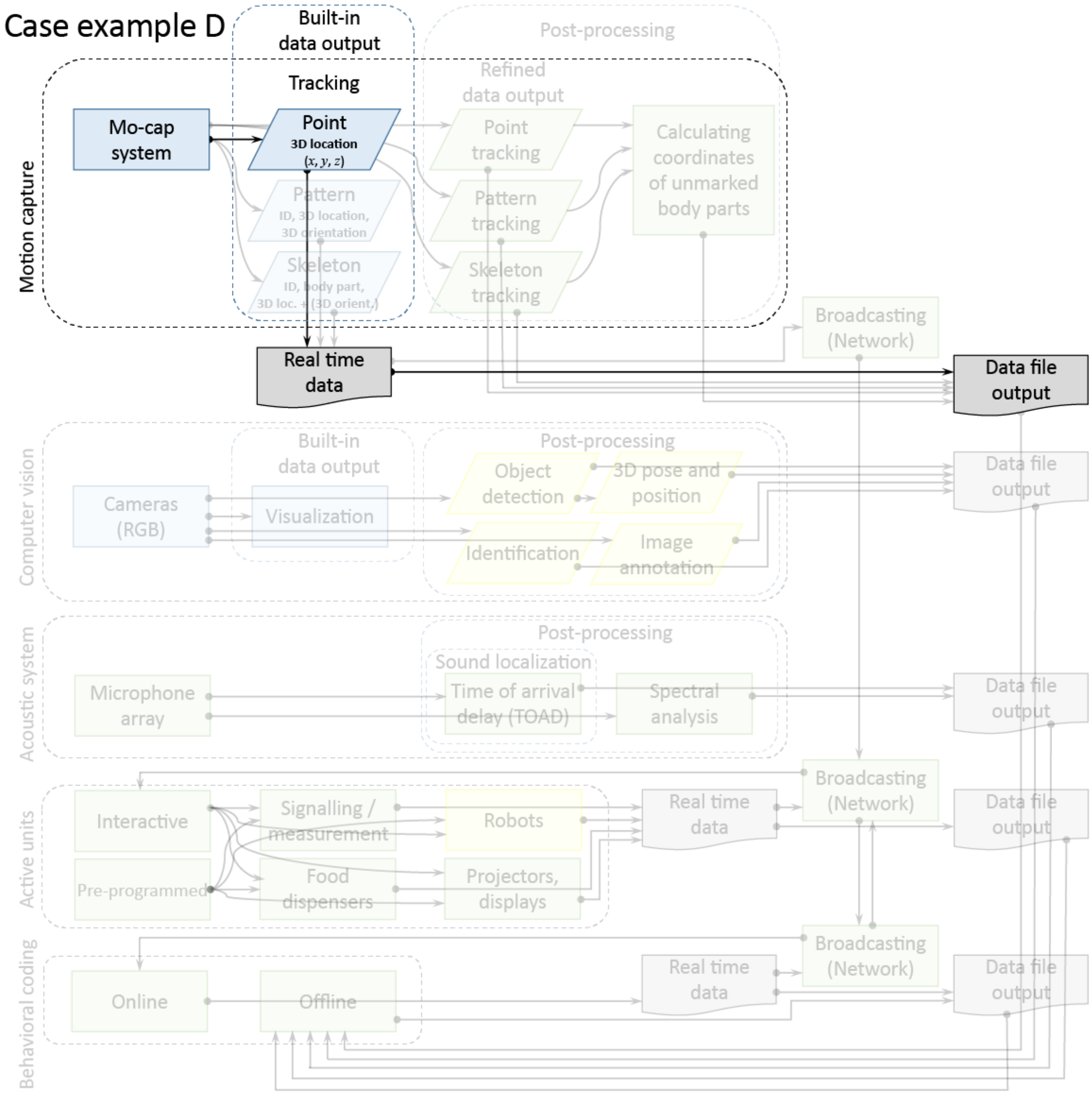

Fig. S9. Components and their connections used in Case example D. For detailed description see Fig. S5.

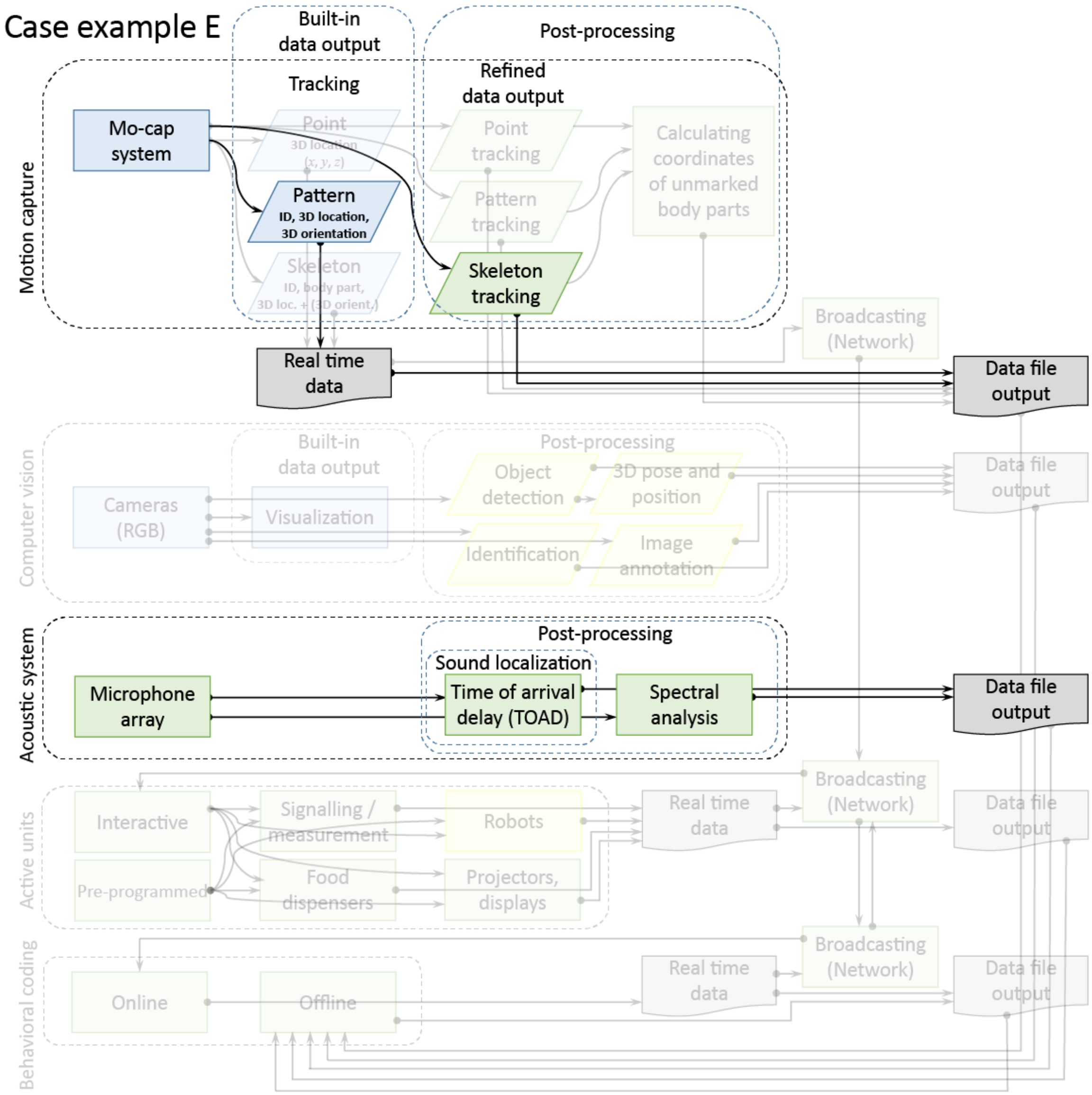

Fig. S10. Components and their connections used in Case example E. For detailed description see Fig. S5.

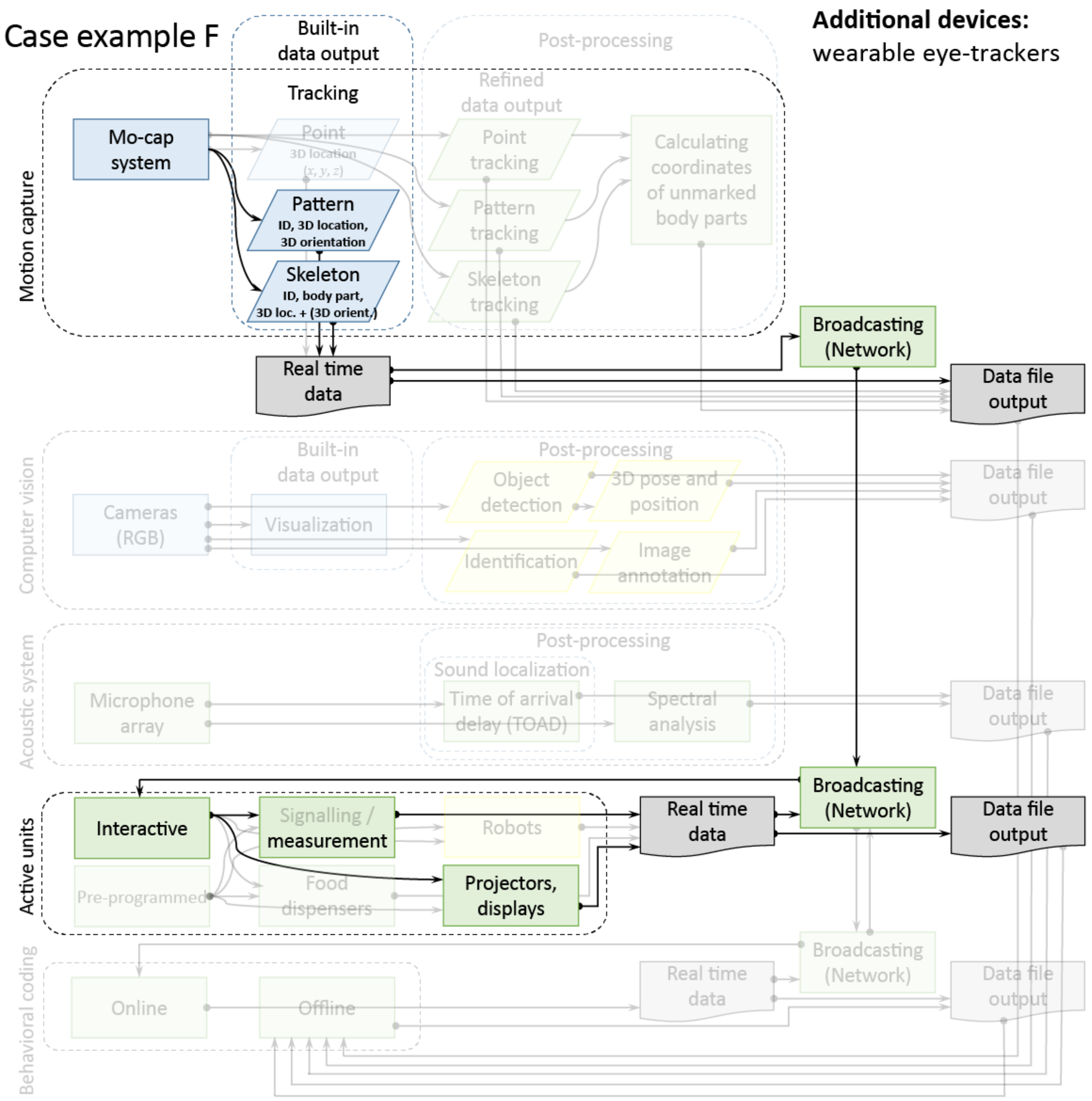

Fig. S11. Components and their connections used in Case example F. For detailed description see Fig. S5. Additional devices used were wearable eye-trackers to provide real-time data shown visualized on a large display.

A

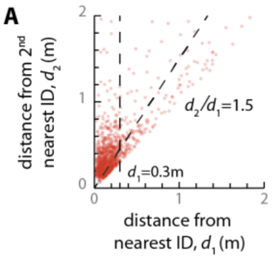

B

	localized sounds					relative occurrence			
d_1	≤0.3m		>0.3m			≤0.3m		>0.3m	
d_2/d_1	≥1.5	<1.5	≥1.5	<1.5	Sum	≥1.5	<1.5	≥1.5	<1.5
day 1					271	61%	30%	3%	7%
day 2					236	63%	15%	6%	16%
day 3	228				333	68%	19%	6%	6%
day 4					305	45%	11%	11%	33%
	→ID					→ID			

	counts				relative occurrence			
**C** localized calls	day 1	day 2	day 3	day 4	day 1	day 2	day 3	day 4
foraging area	0	0	8	5	0%	0%	14%	9%
perching area	67	3	47	46	94%	75%	82%	85%
other	4	1	2	3	6%	25%	4%	6%
Sum	71	4	57	54	71	4	57	54
**D** with ID								
foraging area	0	0	2	0	0%	0%	6%	0%
perching area	34	1	29	27	97%	100%	91%	100%
other	1	0	1	0	3%	0%	3%	0%
Sum	35	1	32	27				
**E** without ID								
foraging area	0	0	6	5	0%	0%	24%	19%
perching area	33	2	18	19	92%	67%	72%	70%
other	3	1	1	3	8%	33%	4%	11%
Sum	36	3	25	27				

**F** behavioral categories	total time (s)				relative occurrence			
	day 1	day 2	day 3	day 4	day 1	day 2	day 3	day 4
foraging	2996	21723	13478	14057	4%	30%	21%	22%
perching	64271	48548	49485	47247	91%	67%	76%	75%
flight	3575	1846	1884	1571	5%	3%	3%	2%
Sum	70842	72117	64847	62875	70842	72117	64847	62875

Fig. S12. Additional analysis for Case study A. (**A**) Assigning sound to individuals by using their motion capture data, and the estimated time and location of the acoustic source. Shown are the distances from the sound source to the nearest (d_1) versus the second nearest (d_2) individuals. Sounds were assigned to an individual, if the nearest bird was closer than 30 cm ($d_1 \leq 0.3$ m), and the second nearest bird was at least 50% more distant than the nearest one ($d_2/d_1 \leq 1.5$; the two criteria are depicted by the dashed lines). (**B**) The number and relative occurrence of localized sounds falling in 4 categories (defined by the 2 criteria described at **A**) throughout the 4 days, showing that around 60% percent of all sounds can be matched to an ID (first column). Note that the increasing relative occurrence of $d_1 > 0.3$m is probably caused by the individuals who damaged their backpacks. (**C-E**) Occurrences (and relative frequencies) of localized calls (**C**) that we could be assigned to an individual (**D**) or we could not (**E**) based on the closest marker pattern. Numbers and the length of the bars show calls at foraging and perching during the 2 hours period for each day. (**F**) We carried out behavioral classification using mo-cap data only to evaluate the time spent in different behaviors (foraging, perching, and flight) pooled for all individuals, during the 2 hours periods for each day.

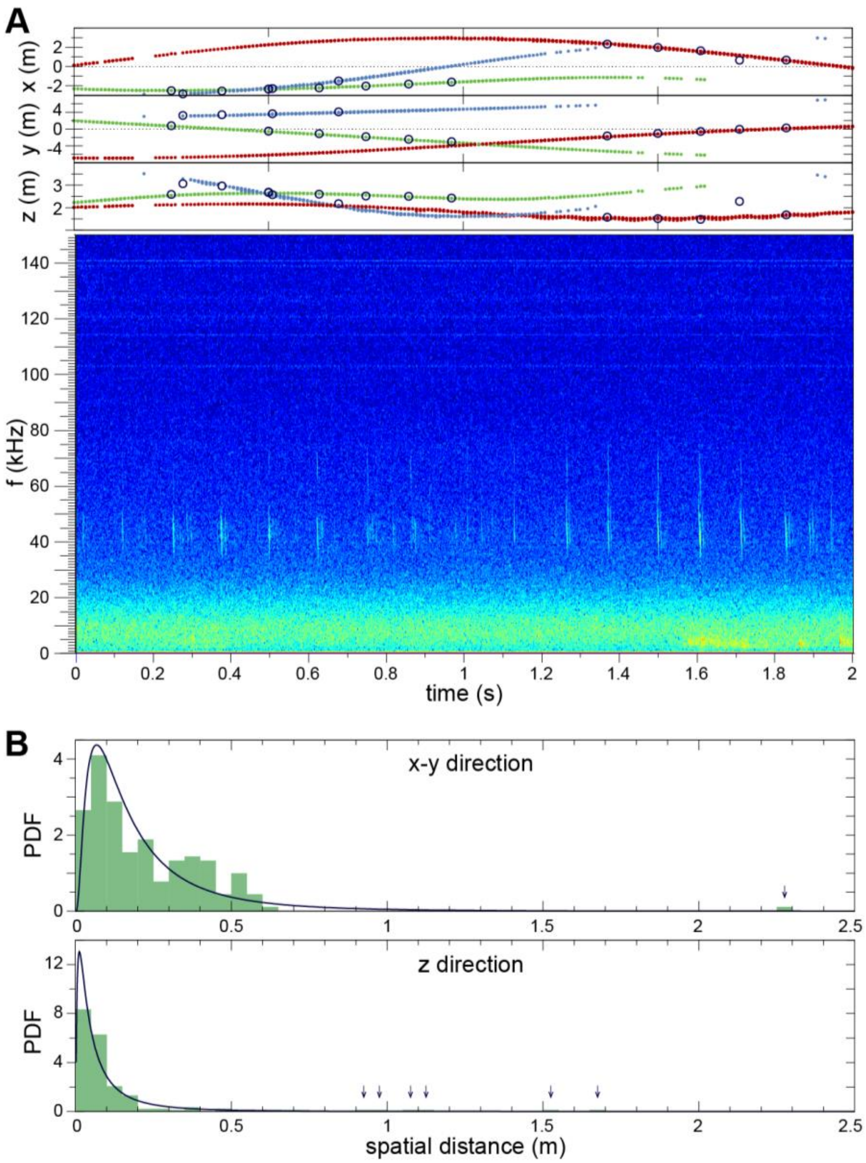

Fig. S13. Acoustic signal and estimation of accuracy for the acoustic localization. (**A**) X, Y, Z coordinates of acoustic localizations (black circles) overlaid on top of the mo-cap tracking (red, green and blue dots) for 3 bats (as shown on main Figure 9E). Here the spectrogram shows the full frequency range recorded from a single microphone channel (located in the center of the ceiling). (**B**) Distribution of distance between spatial coordinates of the localized echolocation clicks and the nearest mo-cap marker location shown as probability density function (PDF) by the green histogram for horizontal (x-y, top) and vertical (z, bottom) direction. Black curves show a lognormal fit to the data, for x-y: mean = 0.15 m, SD = 2.45 m; for z: mean = 0.05 m, SD = 3.30 m. Occasional large values are marked with arrows for better visibility. Note that this serves as a proxy for accuracy of the acoustic localization, as the nearest mo-cap marker is either on its back or on its wrist (if recorded), but not at the exact location where the sound is produced.

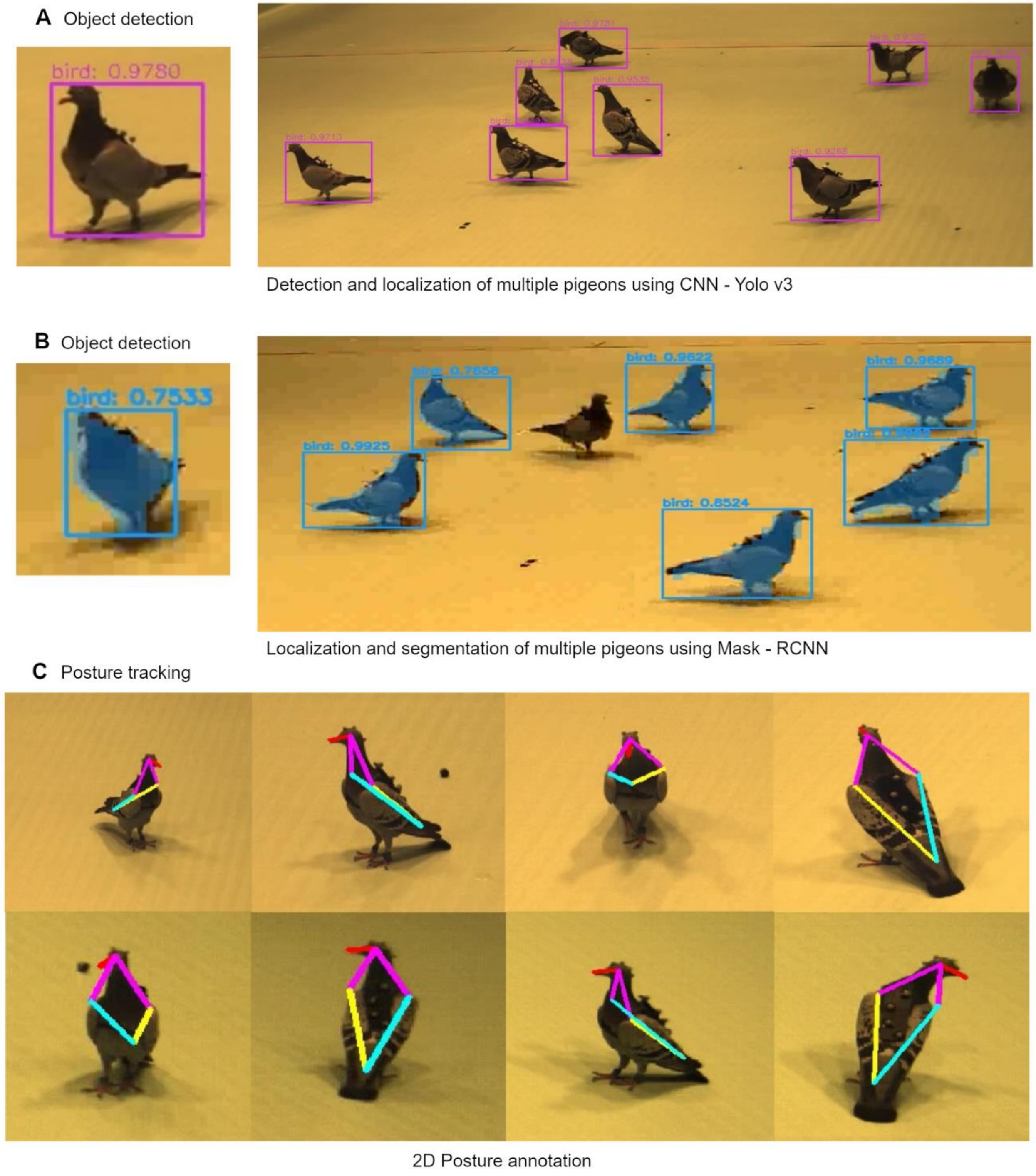

Fig. S14. Marker-less video tracking with machine learning.

(**A-B**) The figure shows different state of the art computer vision methods applied to the dataset obtained from case study B. These methods use supervised training approach and the annotations required for training such algorithms can be obtained directly from our setup by projecting the 3D mo-cap marker tracking results to the image space. (**C**) Complex annotation of 2D posture obtained with our setup.

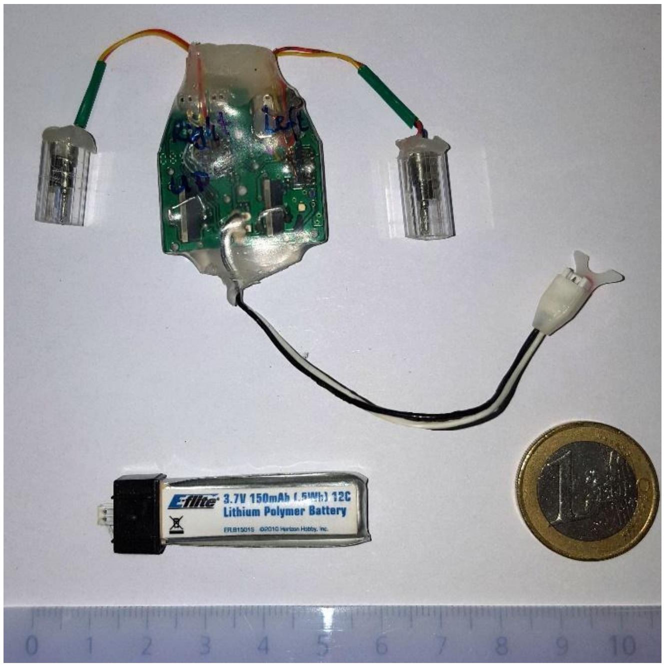

Fig. S15. Haptic feedback (vibration signaling) device used in case study C.

On the top is the central board with the electronics including the RC receiver. The two vibration motors are connected by thin wires to the central board. The electronics are sealed with a heat-shrink tube. The battery (on the bottom) can be connected to the connector on the right side of the photo.

Supplementary Tables

Table S1. Overview of sensors installed in the SMART-BARN with vital specifications.

**Modality**	**Motion capture (IR)**	**Video Camera**	**Acoustic**
Sensor type	Infrared camera	RGB camera	Microphone
No. of sensors	30	6	30
Primary feature	3D marker tracking	Video recording	Acoustic recording
Tracking options	Passive: Retro reflective markers (3D)<br>5 mm – 12 mm<br><br>Active: LED markers	Marker-less 2D/3D (custom)	3D sound localization (custom)<br><br>Active markers: On-board (ultrasonic) speakers
Calibration protocol	Multi-camera calibration (Vicon)	Multi-camera calibration (Vicon)	Custom
Output	3D position<br>6-DOF pose<br>Joint angles	Video	Audio
Operating frequency	Vero up to 330 Hz<br>Vantage up to 420 Hz	Vue up to 100 Hz	100-130 Hz
Live data & Interface	Yes/LAN	No	No
Data processing software	Vicon Tracker,<br>Vicon Nexus	Vicon Nexus,<br>Custom	Custom
Sync options	Hardware trigger	Hardware trigger	Custom
Precision	< 1 mm	9 mm (*40*)	~ 10 cm

Table S2. Overview of three most basic configurations offered with mo-cap system for setting up tracking.

	**Point tracking**	**Pattern tracking**	**Posture**
Output	3D position (*X*, *Y*, *Z*)	3D position (*X*, *Y*, *Z*), 3D orientation (*RX*, *RY*, *RZ*)	3D position (*X*, *Y*, *Z*), 3D orientation (*RX*, *RY*, *RZ*), Joint angles
Tracking constraint	Visibility in at least 2 views	Requires sufficient distance between markers (min. 2 cm)	Predefined markers and patterns required per limb
Maintains identity	No (keeps identity only for continuous segments)	Yes	Yes
Post processing	Software + programmable	Software + programmable	Software + programmable
Limitations	Occlusion	Self-occlusion, Symmetric design	Occlusion

Supplementary Movie Legends

Movie S1. Video montage for Case study A.

(**Clip 1**) Visualization of the tracking system display with motion capture tracking (top left) together with the top-down view (right side) and side view (bottom left) from the video cameras during an experiment with starlings (using Vicon Nexus software and user interface).
(**Clip 2**) Visual-only video from the top-down camera positioned over the perches showing starlings wearing backpacks with marker patterns to allow for IR motion capture tracking.
(**Clip 3**) Visualization of IR tracking-only data in the Vicon interface (Vicon Tracker) of each individuals' marker patterns before post-processing. After post-processing the tracks and the identification of the individuals become almost flawless (as shown in the next clip). The perches are located on the right side from where birds fly to the foraging boxes in the center of the area.
(**Clip 4**) 3D visualization of the tracking data after post-processing (using custom-written visualization), together with the 3D positions of the localized sound sources (calls). The locations of the individuals are indicated by black dots, and their path from the last 5 seconds is shown by a color scale white-yellow-red-black. Acoustic localizations are shown with blue dots, appearing at the time of the origin of the sound source, and slowly shrinking for visualization purposes. To support the 3D visualization of the data, positions are also shown on each plane as gray shadows. The view is matching the view of Figure 3F and 5A for easy comparison. The clip starts with a playback speed matching real time, but then it is sped up by a factor of 10 to show a larger variety of behaviors.

Movie S2. Video montage for Case study B.

(**Clip 1**) A pigeon flock during free foraging is shown by 3 cameras - an overhead view (top) and 2 side-facing cameras providing stereo view (bottom). The videos are overlaid with the motion capture data (using Vicon Nexus). Color indicates the identity of the pigeons.
(**Clip 2**) Visualization of the raw tracking data (head and body patterns) using Vicon Tracker. This is an example of the high precision that can be achieved by the SMART-BARN's motion capture, which is only available within around 30-60 minutes after calibration. After that a new calibration is necessary, but if that is not possible, high-quality tracking data can be achieved by post-processing.
(**Clip 3**) Synchronized view of the side-facing (top left) and overhead (top right) cameras and the 3D visualization of the heads of the pigeons and projection of the foveas to indicate their gaze (bottom center). Green and blue lines demonstrate the projection of the foveas of the right and left eyes, respectively. Red lines show the projection of the red area facing forward.
(**Clip 4**) Synchronized view of a video recording by a side-facing camera (bottom right), and virtual views (generated after the trial using custom-written script in Blender) of 2 pigeons foraging. The virtual view (bottom left) shows a reconstruction of the scene using a fixed camera view with animated 3D mesh objects representing the pigeons (wings and legs are not animated as they were not tracked). To visualize the point of view from a pigeon's perspective (on top), we show the scene from a virtual camera (wide-lens, facing forward) mounted on the head of the pigeon and following the movement and rotation it's head using the motion capture data.

Movie S3. Video supplement for Case study C.

Synchronized view of a side-facing camera (bottom left) and visualization of the motion capture data (top view) during a trial with a pigeon wearing the haptic feedback device. The visualization indicates the location of the 3 seed dispenser (numbered as 0, 1 and 2), the location of the pigeon (yellow dot) and its path from the last minute color-coded by time.
After a feeder was reached, a new target was assigned randomly, as indicated by the "X". The haptic feedback device vibrated on one shoulder of the pigeon to signal a left or a right turn shown with red and blue dots, respectively. When the correct (target) seed dispenser was reached, it was activated as shown by a green dot. Due to constrains in the field of view of a single camera, only seed dispenser 0 (at the back) and 1 (in the foreground) is visible on the camera stream. Seed dispenser 2 located on the left, just outside of the field of view of this camera (although recorded by another side-facing camera just not shown here for simplicity).

Movie S4. Video supplement for Case examples D, E and F.

(**Clip 1**) 3D visualization of the tracking data of 2 hawkmoths (Case example D; using custom-written visualization software). To support the 3D visualization, the positions are also shown on each plane as orthographic projections (purple, green, blue). The playback speed is five times real time, to allow presentation of a longer time segment. A photo of an individual with the mo-cap markers is shown in the bottom right.
(**Clip 2**) Photo of a bat (Case example E) equipped with markers overlaid with the reconstructed skeleton that captures the motion of its wings (with red and blue dots). Video shows the 3D visualization of the tracking data after post-processing with the resulting skeleton, matching the locations as shown on the photo. The playback speed is ¼ times real time to allow presentation of the data recorded at 100 frames per second.
(**Clip 3**) 3D visualization of 4 bats (although only 3 of them were flying together at the same time). Visualization details match Clip 1. The playback speed matches real time. Not all of them were marked on their wings.
(**Clip 4**) Live video (captured on a handheld device) showing detailed tracking of humans (Case example F) in our setup that can be used for similar-sized vertebrates as well. Test subjects were wearing specialized eye-tracking glasses that allowed reconstruction of their gaze in real time as indicated by the screen at the back. Such measurements on humans can be simultaneously performed while doing real-time tracking of animals as shown in the other case studies, for example to study animal-human interactions.
(**Clip 5**) Visualization of the tracking data for the previous clip. Paddles and glasses are highlighted, and red lines depict the gaze.
